# Molecular profile of 5-fluorouracil pathway genes in colorectal carcinoma

**DOI:** 10.1186/s12885-016-2826-8

**Published:** 2016-10-12

**Authors:** T. Kunicka, P. Prochazka, I. Krus, P. Bendova, M. Protivova, S. Susova, V. Hlavac, V. Liska, P. Novak, M. Schneiderova, P. Pitule, J. Bruha, O. Vycital, P. Vodicka, P. Soucek

**Affiliations:** 1 Department of Toxicogenomics, National Institute of Public Health, Prague, Czech Republic; 2 Third Faculty of Medicine, Charles University, Prague, Czech Republic; 3 Biomedical Centre, Medical School Pilsen, Charles University in Prague, Pilsen, Czech Republic; 4 Department of Molecular Biology of Cancer, Institute of Experimental Medicine, Czech Academy of Sciences, Videnska 1083, 142 00 Prague 4, Czech Republic; 5 Deparment of Surgery, Teaching Hospital and Medical School Pilsen, Charles University in Prague, Pilsen, Czech Republic; 6 Department of Surgery, General University Hospital in Prague, First Medical Faculty, Charles University, Prague, Czech Republic; 7 Toxicogenomics Unit, National Institute of Public Health, Srobarova 48, 100 42 Prague 10, Czech Republic

**Keywords:** Colorectal carcinoma, 5-fluorouracil, Methylation, Expression, Prognosis

## Abstract

**Background:**

This study addresses involvement of major 5-fluorouracil (5-FU) pathway genes in the prognosis of colorectal carcinoma patients.

**Methods:**

Testing set and two validation sets comprising paired tumor and adjacent mucosa tissue samples from 151 patients were used for transcript profiling of 15 5-FU pathway genes by quantitative real-time PCR and DNA methylation profiling by high resolution melting analysis. Intratumoral molecular profiles were correlated with clinical data of patients. Protein levels of two most relevant candidate markers were assessed by immunoblotting.

**Results:**

Downregulation of DPYD and upregulation of PPAT, UMPS, RRM2, and SLC29A1 transcripts were found in tumors compared to adjacent mucosa in testing and validation sets of patients. Low RRM2 transcript level significantly associated with poor response to the first-line palliative 5-FU-based chemotherapy in the testing set and with poor disease-free interval of patients in the validation set irrespective of 5-FU treatment. *UPP2* was strongly methylated while its transcript absent in both tumors and adjacent mucosa. *DPYS* methylation level was significantly higher in tumor tissues compared to adjacent mucosa samples. Low intratumoral level of *UPB1* methylation was prognostic for poor disease-free interval of the patients (*P* = 0.0002). The rest of the studied 5-FU genes were not methylated in tumors or adjacent mucosa.

**Conclusions:**

The observed overexpression of several 5-FU activating genes and DPYD downregulation deduce that chemotherapy naïve colorectal tumors share favorable gene expression profile for 5-FU therapy. Low RRM2 transcript and *UPB1* methylation levels present separate poor prognosis factors for colorectal carcinoma patients and should be further investigated.

**Electronic supplementary material:**

The online version of this article (doi:10.1186/s12885-016-2826-8) contains supplementary material, which is available to authorized users.

## Background

Colorectal carcinoma (OMIM: 114500) is the third most common malignancy and the fourth cause of cancer-related deaths in the adult population worldwide, with the highest incidence recorded in Central Europe [1, 2].

Colorectal cancer treatment consists of surgical removal of the tumor and, based on disease characteristics, of chemo- and or radiotherapy. 5-Fluorouracil (5-FU) is widely used drug in the first-line therapy of colorectal cancer [3]. Over 80 % of administered 5-FU dose is rapidly degraded [4] and only 1–3 % is converted into its active metabolite fluorodeoxyuridine monophosphate (FdUMP [5],). FdUMP then inhibits thymidylate synthase (TYMS, OMIM: 188350) and blocks deoxythymidine triphosphate (dTTP) synthesis. Subsequent dTTP depletion triggers “thymineless” death [6]. TYMS is considered as a potential prognostic marker for colorectal cancer. Recent studies have shown that overexpression of TYMS transcript predicts poor outcome in colorectal cancer patients [7, 8]. However, another contemporary study has not confirmed these observations as intratumoral TYMS transcript level was not predictive in patients with colorectal cancer of stage II and III [9].

Several studies have indicated potential prognostic or predictive role of 5-FU metabolizing enzymes expression for resistance to the treatment of colorectal cancer. Colorectal cancer patients with low protein expression of 5-FU inactivating enzyme dihydropyrimidine dehydrogenase (DPYD, OMIM: 612778) exhibited a longer survival after 5-FU-treatment than those with high levels [10]. Likewise, high DPYD transcript level was associated with poor outcome of stage IV colorectal cancer patients [11]. High thymidine phosphorylase (TYMP, OMIM: 131222, 5-FU activating enzyme) transcript level was associated with significantly better disease-free survival (DFS) following oral administration of 5-FU in stage III colorectal cancer patients [12].

The resistance of the tumor cells towards 5-FU is substantially modulated by the transport mechanisms. Especially solute carrier transporter 29A1 (SLC29A1, OMIM: 602193) plays a crucial role in cellular uptake of nucleoside drugs such as cytarabine, gemcitabine, or 5-FU [13]. Results of a recent small scale functional study suggested that high SLC29A1 mRNA levels in colorectal cancer tumor tissue correlate with poor clinical response to 5-FU [14].

In this study we aimed to address importance of gene expression and methylation profile of 15 5-FU genes in tumor and adjacent bowel mucosa tissues of colorectal cancer patients for the patient’s prognosis and the response to 5-FU. Genes were selected from literature and PharmGKB database based on functional evidence from 5-FU pharmacokinetics data (https://www.pharmgkb.org/). Protein expression of two most relevant candidate markers was assessed as another chain underlying 5-FU mode of action.

## Methods

### Studied patients and collection of biological specimen

**Fig. 1:**
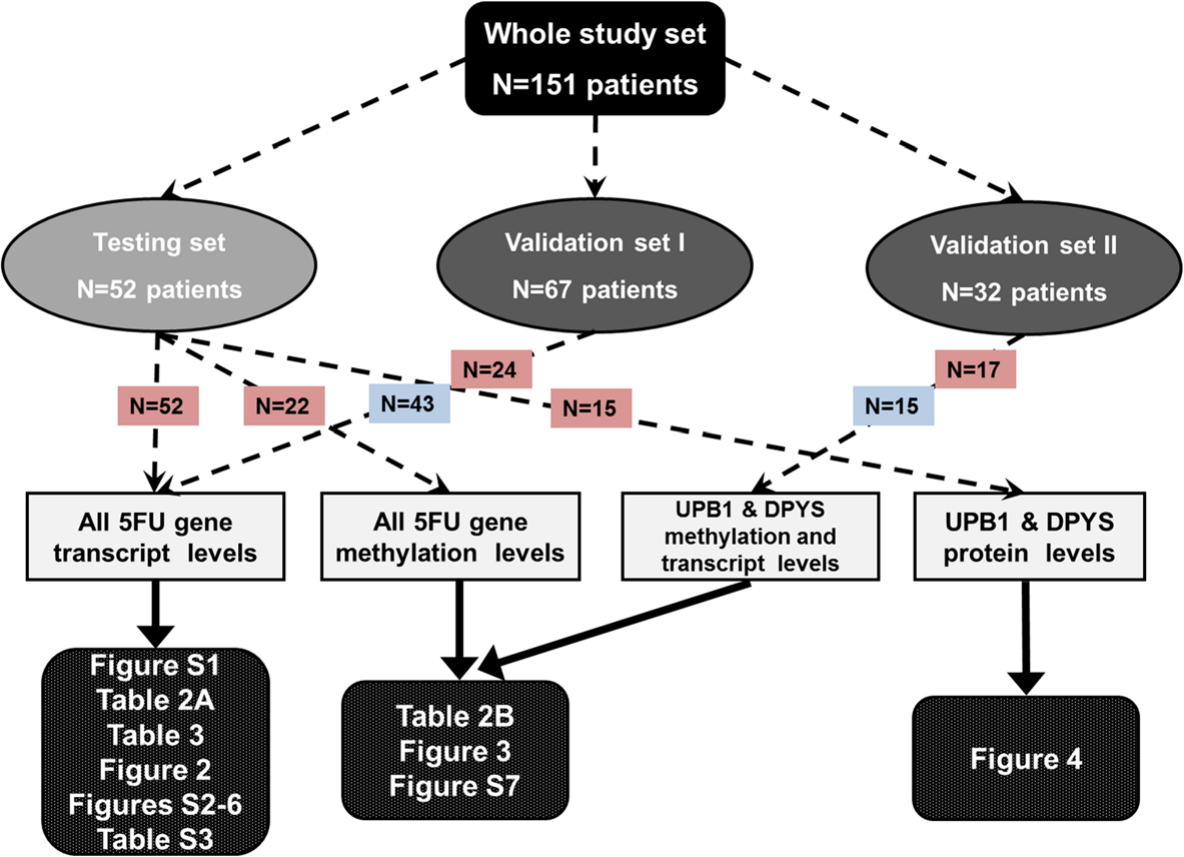
Flow chart of the study. Samples flow and experimental data are displayed by dashed lines and statistical analyses by solid lines. Numbers of 5-FU treated patients in red rectangles and untreated patients in blue rectangles

Tumor tissue and adjacent non-neoplastic mucosa samples were obtained from total of 151 patients with sporadic colorectal cancer (C18-21 according to ICD-10) diagnosed at the Department of Surgery and Oncology, Teaching Hospital and Medical School in Pilsen, and General Teaching Hospital in Prague between January 2008 and November 2011. From 151 patients, 146 paired tissue samples (tumor and control mucosa), four tumors, and one mucosa sample were taken for analyses (for study flow chart, see Fig. 1). Native tissue samples were collected as described elsewhere [15, 16].

Patients represented three groups – testing set (stage II-IV, *n* = 52) for gene and protein expression and methylation analysis, validation set I (stage II, *n* = 67) for gene expression analysis, and validation set II (stage II and III, *n* = 32) for gene expression and methylation analysis. The lack of tissue aliquots for simultaneous isolation of RNA and DNA necessitated the use of two different validation sets. All patients in the testing set underwent adjuvant (*n* = 26) or palliative (*n* = 26) chemotherapy regimens based on 5-FU (with added leucovorin and/or oxaliplatin). In the validation sets I and II, 24 and 17 patients were treated by such chemotherapy regimens, respectively (Table 1 and Fig. 1).

**Table 1:** Clinical-pathological characteristics of studied groups of patients

Characteristics	Testing set	Validation set I	Validation set II
	(*n* = 52)	(*n* = 67)	(*n* = 32)
Gender (male/female)	36/16	45/22	19/13
Age at diagnosis^a^	63.9 ± 9.2 years	70.2 ± 9.5 years	70.8 ± 11.2 years
Tumor size (pT)			
pT2	3	-	4
pT3	40	62	24
pT4	9	5	4
Presence of lymph node metastasis (pN)			
pN0	15	67	18
pN1-2	37	-	14
Presence of distant metastasis (pM)			
pM0	26	67	32
pM1	26	-	-
Stage			
UICC II	8	67	18
UICC III	18	-	14
UICC IV	26	-	-
Histological grade (G)^b^			
GI	6	9	6
GII	39	47	19
GIII	7	8	3
Gx	-	3	4
Primary localization			
Colon	26	44	28
Rectosigmoideum	12	9	1
Rectum	14	14	3
Chemotherapy			
5-FU-based	52	24^c^	17
None	-	33^c^	15

Footnotes:

^a^Median ± standard deviation

^b^ *GI* well differentiated, *GII* moderately differentiated, *GIII* poorly differentiated, *Gx* cannot be assessed

^c^Numbers may not add up to 67 of available subjects because of missing data (*n* = 10)

Response to the palliative treatment was evaluated by RECIST criteria [17] based on routine imaging techniques for assessment of tumor mass (computerized tomography with or without positron emission, magnetic resonance or ultrasonography). Increase in tumor mass or the appearance of new lesions in patients with palliative treatment indicated progression and thus poor response to the treatment (PD). Good response to the treatment was defined as a decrease of the number or volume of metastases, i.e., complete or partial response (CR or PR) or stabilization of the disease or (SD). In patients treated by adjuvant therapy after radical surgical resection R0 disease-free interval (DFI) served as a measure of the treatment outcome. DFI was defined as the time elapsed between radical surgical R0 resection and disease recurrence.

Methylation analyses were conducted on 22 tissue pairs from the testing set and on the whole independent validation set II from the General Teaching Hospital, Prague.

### Isolation of total RNA and cDNA synthesis

Total RNA was isolated from frozen tissues using Trizol® reagent (Life Technologies, Carslbad, CA), stored, and characterized for the quantity and quality [18]. Complementary DNA (cDNA) was synthesized using 0.5 μg of total RNA and random hexamer primers with help of RevertAid™ First Strand cDNA Synthesis Kit (MBI Fermentas, Vilnius, Lithuania). Quality of cDNA in terms of DNA contamination was confirmed by PCR amplification of *ubiquitin C* [19].

### Gene expression profiling

Quantitative real-time PCR (qPCR) was performed using ViiA7 Real-Time PCR System, TaqMan® Gene Expression Assays and TaqMan® Gene Expression Master Mix (Life Technologies). Reference genes - *POLR2A* (DNA-directed RNA polymerase II subunit A, OMIM: 180660), *MRPL19* (mitochondrial ribosomal protein L19, OMIM: 611832), *EIF2B1* (eukaryotic translation initiation factor 2B, subunit 1, OMIM: 606686)*,* and *PSMC4* (proteasome 26S subunit, ATPase, 4, OMIM: 602707) - were selected by us earlier [15]. Gene Expression Assays with their characteristics are listed in Additional file 1: Table S1. While samples from the testing set were preamplified using TaqMan PreAmp Master Mix (Life Technologies), cDNA from the validation sets was used for quantification directly without preamplification procedure [20]. For calculating the qPCR efficiency of each assay, a calibration curve from one non-neoplastic sample was prepared (six points, 5-times dilution). The non-template control contained water instead of cDNA.

The qPCR study design adhered to the MIQE Guidelines (Minimum Information for Publication of Quantitative Real-Time PCR Experiments [21]).

Gene expression and clinical data of all samples were submitted to Gene Expression Omnibus (GEO) repository under accession number GSE67111.

### Promoter CpG methylation profiling

To convert unmethylated cytosines to uracils whole genomic DNA was treated with sodium bisulfite using the Epitect Bisulfite Kit (Qiagen, Hilden, Germany) following the manufacturer’s protocol. Promoter region of every gene of interest was determined using Genomatix MatInspector and Genes & Genomes software (Genomatix Software GmbH, Munich, Germany). CpG islands or simple CpG sites were identified by Methyl Primer Express Software v1.0 (Applied Biosystems, Foster City, CA). The same software was used for design of primers specific for sodium bisulfite converted DNA bases. Number of CpGs in the PCR amplicon and equal primer melting temperature (Tm) were taken into consideration in the primer design. Real-time PCR followed by high resolution melting (HRM) was carried out in high-performance Eco Real-Time PCR system (Illumina, San Diego, CA), essentially as described in [16]. PCR was initiated by incubation at 95 °C for 5 min, followed by 50 cycles at 95 °C for 10 s, annealing temperature of specific primers (Ta) for 20 s, and 72 °C for 10 s. Primer sequences, Tm, Ta, length, and numbers of CpGs for each amplicon are listed in Additional file 1: Table S2. HRM thermal profile was set up according to the manufacturer’s recommendations (Qiagen). Fluorescence data were converted into melting peaks by the Eco Software (Illumina, Ver. 3.0.16.0). For each assay, a standard dilution series of EpiTect Control DNAs (Qiagen) was run to assess the quantitative properties and sensitivity of the assay. Fluorescence of each sample was normalized against 100 % methylated DNA control. Methylation data of individual samples were subtracted from calibration curve with positive controls of 100, 75, 50, 25, and 0 % methylated DNA.

### Immunoblotting in human colorectal cancer tissues

Tissue sample pairs from 15 patients and unpaired tumors from two patients were selected based on tissue availability from the testing set and used for immunoblotting. Samples, stored at −80 °C prior to the protein isolation, were grinded by a mortar and pestle, subsequently protein and total RNA were isolated using 50 mM Tris–HCl, 150 mM NaCl, 10 % Triton X-100 buffer. Protein concentration was determined by bicinchoninic acid assay (Thermo Scientific Pierce Protein Research Products, Rockford, IL). Immunoblotting was performed as described in [20, 22]. Briefly, 10 μg of protein was used for separation by sodium dodecyl sulfate polyacrylamide gel electrophoresis (10 %) and transferred onto 0.2 μm Protran nitrocellulose membrane (Whatman, Kent, UK). Primary antibodies against dihydropyrimidinase (DPYS, OMIM: 613326) (dilution 1:4000), beta-ureidopropionase (UPB1 OMIM: 606673) (dilution 1:500) (both from Aviva System Biology, San Diego, CA), β-actin (dilution 1:2000; Sigma-Aldrich, St. Louis, MO) and the corresponding horseradish-peroxidase-conjugated secondary antibodies (dilution 1:10000; Sigma-Aldrich) were employed. Protein bands were visualized with an enhanced chemiluminescence detection system (Pierce Biotechnology) by Fc Odyssey (Licor Biotechnology, Lincoln, NE) and quantified by densitometry (Image Studio software, Licor Biotechnology).

### Statistical analyses

Expression levels of genes were analyzed by ViiA7 System Software (Life Technologies) and statistical analysis was performed using SPSS v16.0 Software (SPSS Inc., Chicago, IL). Fold changes were calculated usig raw cycle threshold (Ct) data by the REST2009 program (Qiagen), which is routinely used for the determination of differences between different types of sample and control groups and considers both normalization to numerous reference genes and reaction efficiencies [23]. Then ratios of Ct values of genes of interest and mean value of reference genes were calculated and used for further statistical analyses. Differences in gene expression or methylation levels between tumor and control tissues were assessed by the nonparametric Mann-Whitney U-test. To evaluate associations of transcript levels with clinical data and other variables (Table 1), nonparametric tests (the Kruskal-Wallis, the Mann-Whitney, and the Spearman’s tests) were used.

DFI was evaluated by the Kaplan-Meier method and the Log Rank test was used for evaluation of the compared subgroups and combined groups of patients. Stage-adjusted analysis was performed by the Cox regression. All *P*-values were calculated from two-sided tests. *P*-values lower than 0.05 were considered statistically significant. The correction for multiple testing was applied according to Bonferroni.

## Results

### Patients’ characteristics

Summary of patient’s characteristics and clinical data from testing and validation sets are presented in Table 1 and the study flow diagram in Fig. 1. Testing set comprised colorectal cancer patients with stages UICC II-IV treated by first-line adjuvant (*n* = 26, UICC II and III) and palliative chemotherapy based on 5-FU (*n* = 26, UICC IV). Testing set served as a hypothesis generating screen and for assessment of protein levels. Validation set I used for validation of gene expression study included patients with UICC II stage (*n* = 67). Part of them was treated by 5-FU-based chemotherapy (*n* = 24). Validation set II used for methylation study consisted of patients with UICC II and III stage (*n* = 32) with 17 patients treated by 5-FU-based chemotherapy. The validation set II served for validation of correlations between DPYS and UPB1 methylation and expression levels and clinical data, mainly DFI. Median DFI of the validation set I was 46 ± 6 months and that of the validation set II was 39 ± 3 months.

### Transcript levels in tumors and non-neoplastic control tissues

Phosphoribosylpyrophosphate amidotransferase (PPAT, OMIM: 172450), uridine monophosphate synthetase (UMPS OMIM: 613891), ribonucleotide reductase M2 (RRM2, OMIM: 180390), and SLC29A1 transcripts were consistently overexpressed in tumors compared to adjacent mucosa in both testing and validation I sets (except UMPS, all passed the correction for multiple testing, Table 2a, Additional file 1: Table S1). On the contrary, DPYD was downregulated in tumors compared to adjacent mucosa (*P* < 0.001, both sets).

**Table 2:**
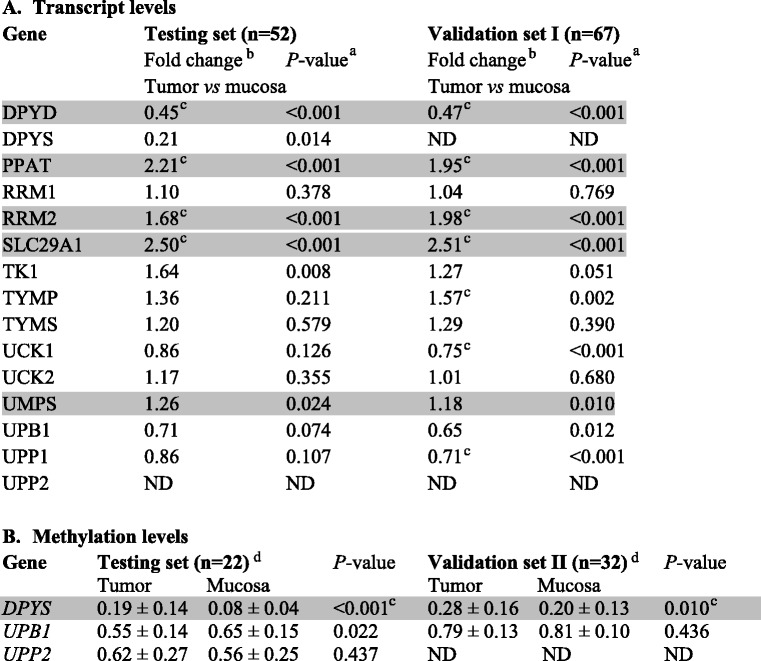
Differences in transcript (A) and methylation (B) levels between tumor and adjacent mucosa tissues of colorectal cancer patients

Footnotes:

^a^Analyzed by the Mann-Whitney test

^b^Fold changes calculated by the REST2009 program

^c^Results, which passed correction for multiple testing

^d^Mean ± standard deviation of percentage of sample methylation normalized to positive control (Methods)

*ND* not determined

Results from the testing set that have been confirmed in the validation set of patients are depicted in grey

### Associations of transcript levels with clinical data of patients

We first tested associations between gene expression levels and therapy response of stage IV patients. Patients from the testing set with poor response to the first-line palliative treatment with 5-FU-based regimens had significantly lower expression of UMPS, ribonucleotide reductase M1 (RRM1, OMIM: 180410), and RRM2 in adjacent mucosa (*n* = 26; *P* = 0.024, *P* = 0.014, and *P* = 0.038, respectively; none passed the correction for multiple testing) than good responders (Table 3). Stage IV patients were excluded from subsequent survival analyses due to the metastatic character of their disease, which strongly modifies their prognosis.

**Table 3:** Differences in transcript levels in colorectal mucosa between poor and good responders to 5-FU-based chemotherapy. Transcript levels of 5-FU pathway genes were compared in mucosas of patients in the testing set divided into groups of poor responders (*n* = 13) and good responders (*n* = 13) to the first line chemotherapy regimens based on 5-FU

Gene	Expression level in poor responders vs. good responders		
	Fold difference^b^	Standard error^b^	*P*-value^a^
DPYD	0.76	0.31–1.69	0.259
DPYS	0.91	0.10–6.89	0.434
PPAT	0.88	0.20–3.63	0.086
**RRM2**	**0.31**	**0.11–1.46**	**0.038**
**RRM1**	**0.59**	**0.22–1.18**	**0.014**
SLC29A1	0.76	0.17–2.73	0.369
TK1	0.87	0.18–3.66	0.157
TYMP	0.56	0.12–2.46	0.130
TYMS	0.82	0.15–3.77	0.121
UCK1	0.85	0.23–1.96	0.369
UCK2	0.74	0.17–2.42	0.681
**UMPS**	**0.68**	**0.23–1.21**	**0.024**
UPB1	0.91	0.26–2.85	0.479
UPP1	0.61	0.16–2.10	0.106

Footnotes:

^a^Analyzed by the Mann-Whitney test

^b^Fold changes and standard error calculated by the REST2009 program

Significant results in bold

**Fig. 2:**
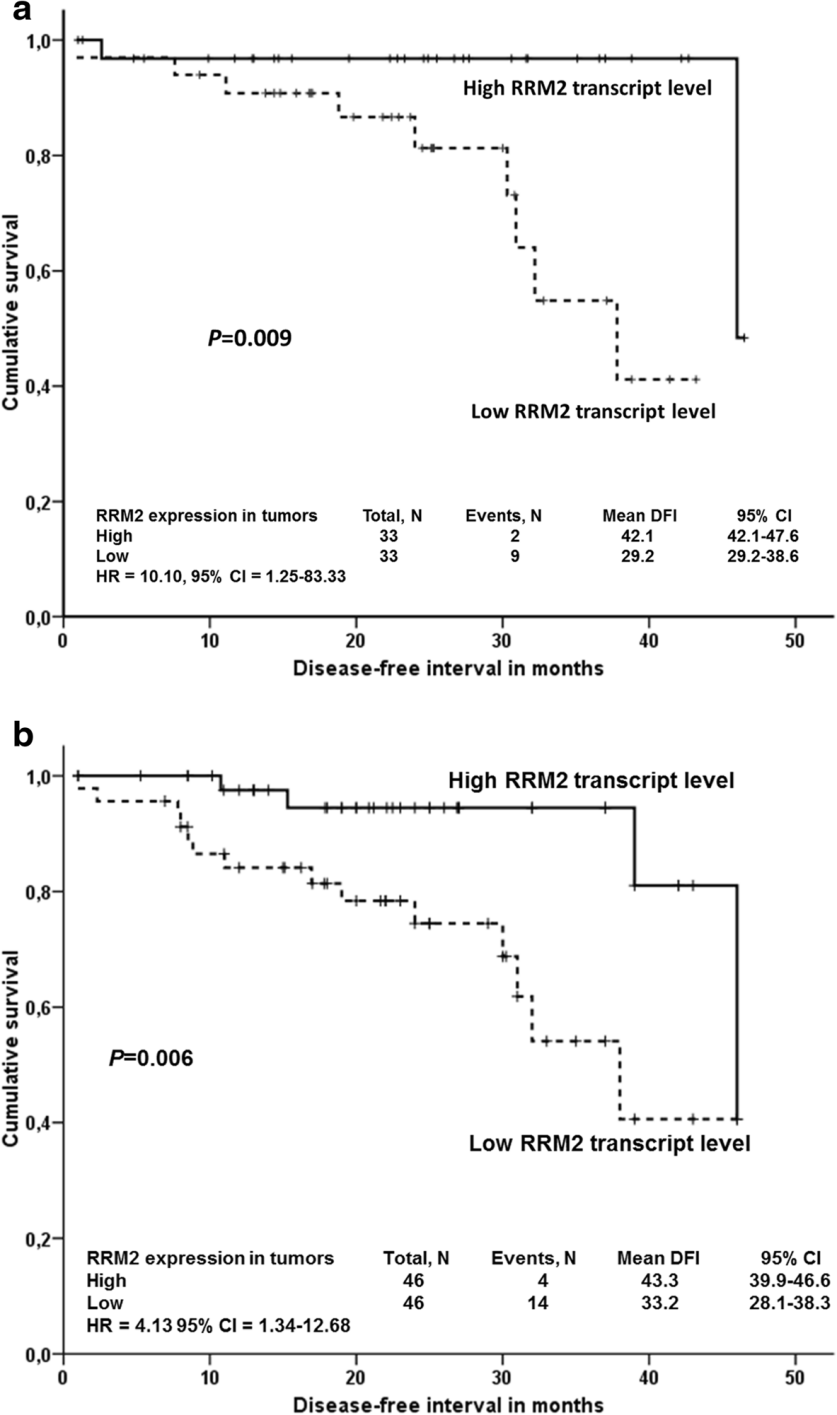
Association between RRM2 transcript levels and DFI of colorectal cancer patients. Kaplan-Meier survival curves were plotted for patients (*n* = 66, one patient was lost to follow up) from the validation set I (**a**) or combined testing and validation I sets (*n* = 92) (**b**). Patients were divided into two groups according to the median of transcript levels in tumors. Dashed line represents the group with lower transcript levels, solid line the group with higher transcript levels than median. Differences between groups were compared using Log-rank test. All genes have been analyzed, but to retain concise style only significant association is reported. HR = hazard ratio, 95 % CI = 95 % confidence intervals for stage-adjusted analyses

For DFI analyses, transcript levels were first divided by their median separately in testing and validation set I and for the combined analysis these data were put together to eliminate raw data differences between sets. Significance of RRM2 gene expression for prognosis of colorectal cancer patients was further corroborated in the validation set I, where patients with intratumoral RRM2 transcript level higher than median had significantly longer DFI compared to patients with levels below the median (*n* = 66, *P* = 0.009, did not pass the correction for multiple testing, Fig. 2a, the rest of results in Additional file 1: Figure S2). A non-significant association in the same direction, was observed in the testing set (*n* = 26, Additional file 1: Figure S3). Analysis of the combined testing and validation I sets supported the findings of the validation set I for RRM2 (*n* = 92, *P* = 0.006, did not pass the correction for multiple testing, Fig. 2b, the rest of results provided in Additional file 1: Figure S4). This association was significant also in stage-adjusted analysis by the Cox regression of the combined set (*n* = 92, *P* = 0.013, HR = 4.17, 95 % CI = 1.35-12.50, for all results see Additional file 1: Table S3).

Then the combined set was analyzed in respect to chemotherapy by 5-FU containing regimens (*n* = 50). However, in the combined analysis of 5-FU-treated patients from the testing and validation I sets, neither RRM2 transcript level (*P* = 0.301) nor levels of the rest of genes did significantly associate with DFI (Additional file 1: Figure S5). Stage-adjusted analysis has shown significant association between UPB1 and DFI (*P* = 0.047, HR = 0.25, 95 % CI = 0.06–0.98, for all results see Additional file 1: Table S3), which was not significant in the univariate analysis (*P* = 0.098, Additional file 1: Figure S5).

In DFI analyses of untreated patients (*n* = 32, all stage II from the validation set 1), low level of UPB1 (*P* = 0.026, did not pass the correction for multiple testing) and TYMP (*P* = 0.047, did not pass the correction for multiple testing) significantly associated with worse DFI of patients (Additional file 1: Figure S6).

### Methylation levels in tumors and non-malignant adjacent mucosa, associations with gene expression, and clinical characteristics

Methylation of CpG islands in the regulatory regions of all studied genes was initially studied in 22 pairs of tumor and adjacent mucosa (testing set) and compared with that from the independent validation set II. In the both testing and validation II sets, methylation exceeding the limit of quantitation was detected in *DPYS*, *UPB1*, and uridine phosphorylase (*UPP2*, GeneID: 151531) genes in both tumor and adjacent mucosa samples (Table 2b, DPYS passed the correction for multiple testing). Significantly elevated methylation level of *DPYS* was recorded in tumor tissues compared to adjacent mucosa in both sets (Table 2b). Methylation level of *UPB1* was lower in tumors than in adjacent mucosa in the testing set, but not in the validation set II. No difference in promoter methylation was observed for *UPP2* in the testing set by comparing tumors with non-malignant mucosa.

Methylation levels in promoter regions of *DPYS* or *UPB1* did not correlate with their corresponding transcript levels either in tumors or in adjacent mucosa samples analyzed in both sets. UPP2 transcript expression was below the limit of quantification in both testing and validation II sets suggesting that this gene is completely silenced in colorectal tumors and corresponding adjacent mucosa tissues regardless clinical characteristics.

**Fig. 3:**
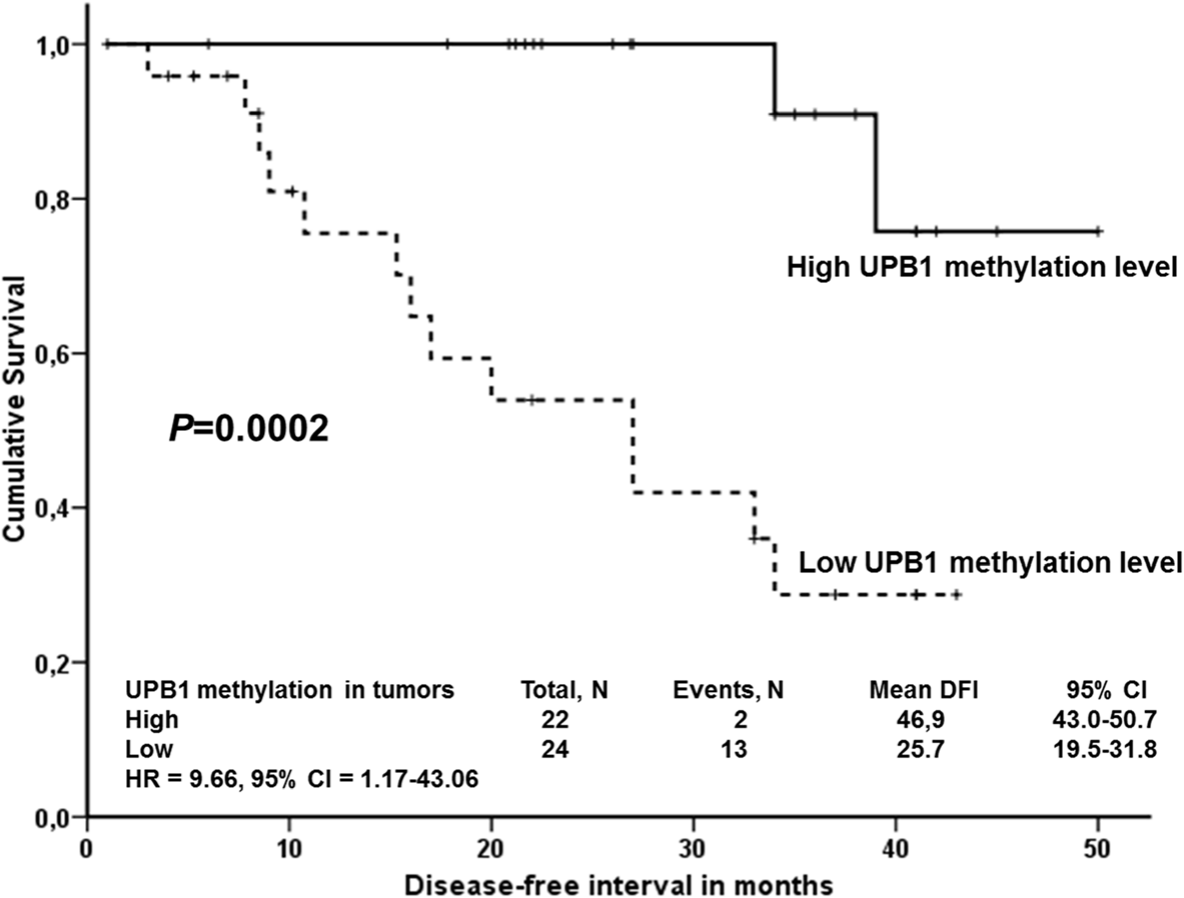
Association between *UPB1* methylation levels and DFI of colorectal cancer patients. Kaplan-Meier survival curves were plotted for patients from the both testing and validation II sets combined (*n* = 46). Seven stage IV patients were excluded and for further 31 patients data on methylation or DFI were not available. Patients were divided into two groups according to the median of intratumoral gene methylation levels. Dashed lines represent the group with lower methylation levels and *solid lines* represent the group with higher levels than median. Differences between these groups were compared using Log-rank test. HR = hazard ratio, 95 % CI = 95 % confidence intervals for stage-adjusted analyses

*DPYS* methylation level was associated with the tumor stage in the testing set (*P* = 0.010, data not shown), but not in the validation set II. Therefore, this association is not further discussed. On the other hand, patients with *UPB1* methylation level below the median had significantly worse DFI than those with the methylation level above the median in both sets evaluated separately (Additional file 1: Figure S7) and combined (*n* = 46, *P* = 0.0002, passed the correction for multiple testing, Fig. 3). This association was significant also in the stage-adjusted analysis by Cox regression of the combined set (*n* = 46, *P* = 0.004, HR = 9.22, 95 % CI = 2.04-41.57).

Combined analysis of *UPB1* methylation in 5-FU treated patients from testing and validation II sets failed to find significant association with DFI (*n* = 32, *P* = 0.653, data not shown). For DFI analyses, patients were divided into two groups according to the median of methylation levels in tumors. Methylation levels of *DPYS* and *UPP2* have not associated with the DFI of patients (*P* > 0.05).

### Protein levels in tumors and adjacent non-malignant mucosa

**Fig. 4:**
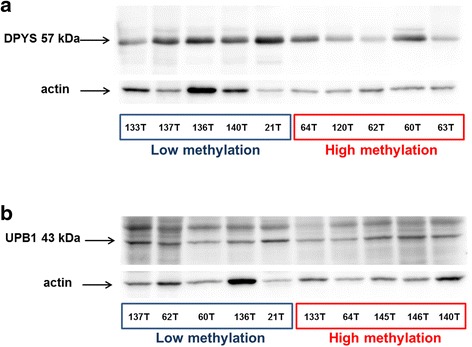
Protein expression of DPYS and UPB1 in tumors of colorectal cancer patients. Protein expression of DPYS (**a**) and UPB1 (**b**) was assessed by immunoblotting with normalization to actin in the representative set of tumors with highest and lowest methylation levels as described in Materials and Methods

DPYS and UPB1 protein levels were analyzed in a subset of the testing set used for the methylation study, enabling an evaluation of the cascade of methylation, gene, and protein expression levels in colorectal cancer samples (Fig. 4). However, DPYS and UPB1 protein levels did not significantly correlate either with their transcripts or methylation levels (*P* > 0.05).

## Discussion

The questions connected with prognostic importance of molecular profile of 5-FU pathway in colorectal cancer remain attractive topics throughout last 15 years. Existing studies offered a plethora of mostly conflicting results. The absence of complex understanding, focused on mechanisms of action underlying the most promising biomarkers precludes their translation into clinical setting. Apparently, the final prognostic scheme will integrate clinical factors, e.g., stage and grade of the tumor with a cascade of molecular markers involving genetic, epigenetic, and phenotypic factors. The present study brings completely new insight into this area by comprehensive molecular profiling of major 5-FU pathway genes.

The present study shows for the first time that only three (*DPYS, UPB1*, and *UPP2*) out of 15 evaluated 5-FU genes, are subject to notable methylation in tumor and adjacent mucosa tissues.

Association of *UPB1* promoter methylation with worse prognosis of colorectal cancer patients, reported here on two independent groups of patients and in the combined set irrespective of 5-FU treatment, poses a completely novel direction in pharmacogenomics of colorectal cancer. UPB1 is an 5-FU inactivating enzyme [24], responsible for degradation of pyrimidine bases (uracil and thymine) and its genetic defect causes severe forms of propionic acidemia [25]. We hypothesized that a high UPB1 expression in tumor cells caused by promoter demethylation could exert a negative impact on the colorectal cancer patients response to 5-FU. However, we did not prove such association in the combined set of 5-FU treated patients and moreover, *UPB1* methylation level did not correlate with either the transcript or the protein levels suggesting that its prognostic role is most probably a complex phenomenon involving some other factors. The lack of such correlation may be explained by a number of effects, e.g., variation in DNA folding in the studied region, regulation of target gene by enhancers/silencers or by other than the followed CpGs or control of gene expression by histone modifications. A more refined screening of CpG methylation in the *UPB1* surrounding area could provide more information about potentially linked epigenetic changes. Moreover, the function of the above mentioned gene may also be modulated by microRNA interference (e.g., hsa-miR-216a, predicted by TargetScan).

From the genetic point of view it is intriguing that recent study reported a strong association between the rs2070474 polymorphism and gastrointestinal toxicity in 5-FU treated cancer patients [26]. It is of interest that this polymorphism lies inside a large CpG island consisting of 98 CpG sites [27] and near to the transcription factor-binding motifs corresponding to a critical regulator of the intestine, the CDX2 (caudal-type homeobox transcription factor 2, OMIM: 600297 [28];). A potential linkage of genetic with epigenetic changes thus should also be considered.

On the basis of our gene expression data we may generalize, that colorectal tumors irrespective of the stage and localization share common downregulation of DPYD and upregulation of PPAT, UMPS, RRM2, and SLC29A1 transcripts. RRM2 and UMPS upregulations and DPYD downregulation in colorectal tumors comply with the previous study [29].

**Fig. 5:**
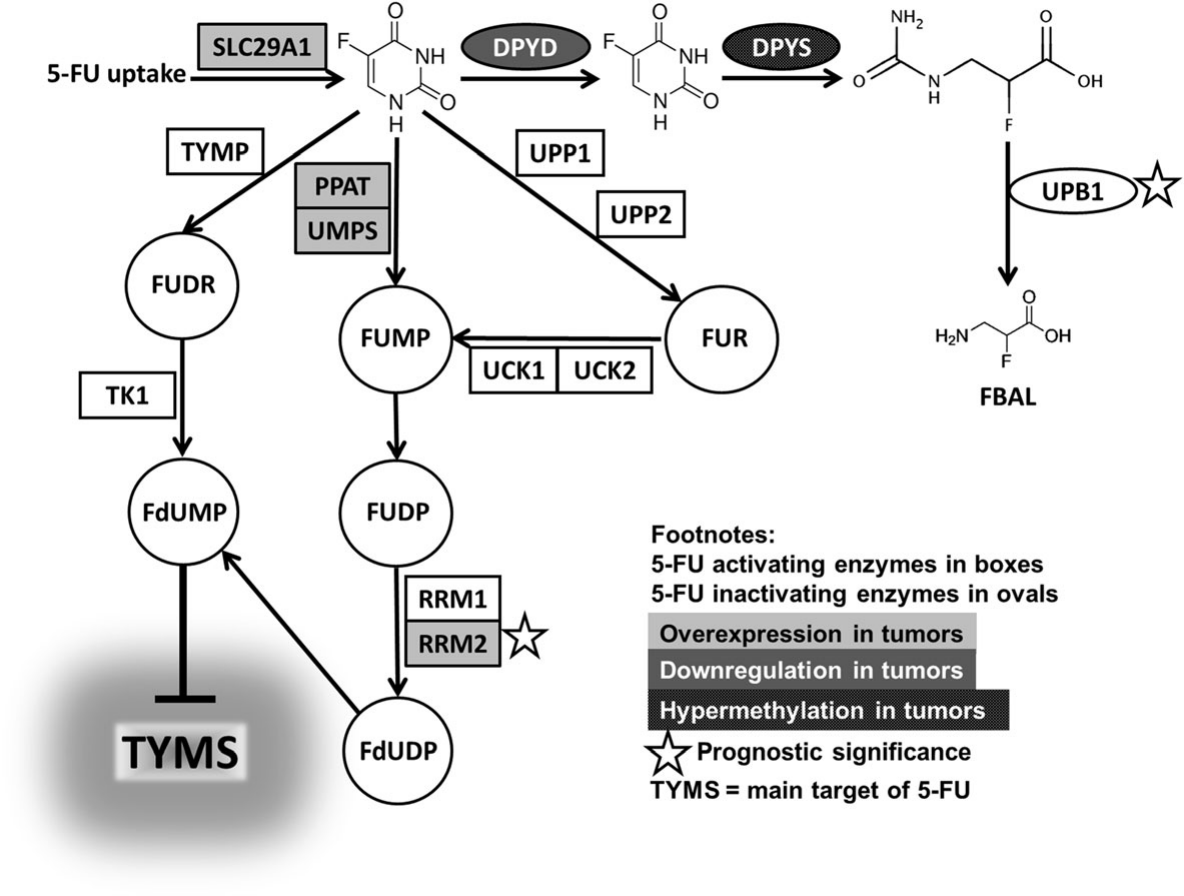
5-FU pathway genes evaluated by this study (adopted from [24])

Interestingly, SLC29A1 was recently suggested as potential co-determinant of clinical response to 5-FU [14] and its upregulation demonstrated by the present study further underpins the potential for targeted therapy of colorectal cancer. On the basis of gene expression profile we may deduce that chemotherapy-naïve colorectal cancer patients have in general favorable expression profile shifted towards 5-FU activation (Fig. 5). A potential change of this profile by chemotherapy or during metastatic process presents another interesting question that needs to be addressed.

Moreover, promoter of the 5-FU inactivating enzyme DPYS was found hypermethylated in colorectal tumors by this study. We thus confirmed the previously published *DPYS* hypermethylation in colon carcinomas (and breast and prostate carcinomas) compared with paired normal tissues from the same patients [30]. Recently, it was reported that differential methylation of *DPYS* (and heat shock 27 kDa protein 1, *HSPB1*, OMIM: 602195 and cyclin D2*, CCND2*, OMIM: 123833) provides independent prognostic information for prostate carcinoma [31]. Based on the present and earlier studies, colorectal cancer-specific complex prognostic model based on gene expression and methylation profile seems to deserve further exploration.

Prognostic significance of low RRM2 transcript level for poor colorectal cancer patient’s outcome observed by the present study contradicts the previously published data. High RRM2 level was poor survival predictor in colorectal cancer patients [32] reflecting the established in vitro ability of RRM2 to enhance cellular invasiveness and genetic instability [33]. We cannot rule out that the qPCR assay for RRM2 employed in the present study also covered the RRM2B (OMIM: 604712) subunit whose protein structure is 80 % identical to RRM2. RRM2B intriguingly exerts opposite activity to RRM2 and its expression associates with a better survival of colorectal cancer patients [34]. On the other hand, RRM2 is 5-FU activating enzyme [24] and thus the result observed by us seems logical from this point of view despite the fact that we have not observed a direct link between prognostic role of RRM2 and 5-FU therapy (perhaps due to the low number of the followed patients). Bearing in mind the issue of study size and publicly available gene expression data, we analyzed the prognostic power of RRM2 expression by SurvExpress [35] tool using data from GSE12945 set (*n* = 947). A borderline significant association towards higher risk of shorter disease-free survival of the patients with lower expression of RRM2 was apparent (*p* = 0.050, Additional file 1: Figure S8).

The present study in line with other authors [9], has not confirmed that overexpression of TYMS protein or transcript predicts poor outcome in colorectal cancer patients [7, 8]. Similarly, the results of studies indicating potential prognostic role of DPYD [10, 11] or TYMP [12] expression for survival of colorectal cancer patients after 5-FU-treatment were not replicated.

The small sample size and small patient’s groups used for DFI analyses, especially of patients treated with 5-FU pose the major limitations of this study. Nevertheless, we compared the methylation profiles with the publicly available database MethHC (Methylation and gene expression in Human Cancer, http://methhc.mbc.nctu.edu.tw) integrating gene expression, methylation, and microRNA expression data from The Cancer Genome Atlas (TCGA) [36]. Our data complies with the results reported by this database, i.e., the highest levels in *UPP2*, *UPB1*, and *DPYS* (the rest of the genes below 25 %) and significantly higher methylation of *DPYS* in tumor compared with mucosa tissues (Additional file 1: Figure S9).

The variability among the patient cohorts could also explain the lack of replication of some results. On the other hand, the use of validation sets helped to achieve more convincing interpretation of the replicated results and where possible the analysis of combined sets increased the study power. The lack of tissue aliquots for simultaneous isolation of RNA and DNA necessitated the use of two different validation sets. This fact precluded us to perform the otherwise preferable combined analyses of both validation sets. Consequently, missing data for comparison of methylation levels with DFI may be seen as a study limitation.

## Conclusions

In this study, we addressed importance of genes involved in the 5-FU pathway for the prognosis of colorectal cancer patients. In conclusion, chemotherapy-naïve colorectal tumors seem to have favorable 5-FU pathway gene expression profile. Additionally, low RRM2 gene expression and *UPB1* methylation level represent treatment-independent poor prognostic factors for colorectal carcinoma patients and should be further investigated in relation to other epigenetic regulation pathways (such as microRNAs) and in a complexity with other relevant systems, such as DNA repair.

## Additional file

Additional file 1: Table S1. Lists TaqMan Gene Expression Assays used in the study. **Table S2** shows sequence of primers and PCR conditions used for promoter CpG methylation profiling. **Table S3** shows results of stage-adjusted Cox regression of associations between transcript levels and DFI of colorectal cancer patients from the combined testing and validation I sets. **Figure S1** depicts 5-Fluorouracil pathway gene expression levels in the studied sets of colorectal cancer patients. **Figure S2** shows results of analysis of associations between transcript levels and disease-free survival of colorectal cancer patients from the validation set I. **Figure S3** shows results of analysis of associations between transcript levels and disease-free survival of colorectal cancer patients from the testing set. **Figure S4** shows results of analysis of associations between transcript levels and disease-free survival of colorectal cancer patients from the combined testing and validation I set. **Figure S5** shows results of analysis of associations between transcript levels and disease-free survival of 5-fluorouracil-treated colorectal cancer patients from the combined testing and validation I set. **Figure S6** shows results of analysis of associations between transcript levels and disease-free survival of untreated colorectal cancer patients from the validation I set. **Figure S7** shows results of analysis of associations between *UPB1* methylation levels and disease-free survival of colorectal cancer patients. **Figure S8** shows analysis of association of RRM2 expression with disease-free survival of colorectal cancer patients based on publicly available GEO database. **Figure S9** shows analysis of methylation profiles of 5-FU pathway genes in human colorectal tumor (red boxes) and mucosa (green boxes) tissues from publicly available MethHC database. (DOC 1916 kb)

## Abbreviations

5-FU: 5-fluorouracil
95 % CI: 95 % confidence interval
CCND2: Cyclin D2
cDNA: Complementary DNA
CDX2: Caudal-type homeobox transcription factor 2
CpG: Cytosine-phosphate-guanine
CR: Complete response
DFI: Disease-free interval
DFS: Disease-free survival
DPYD: Dihydropyrimidine dehydrogenase
DPYS: Dihydropyrimidinase
dTTP: Deoxythymidine triphosphate
EIF2B1: Eukaryotic translation initiation factor 2B, subunit 1
FDR: False discovery rate
FdUMP: Fluorodeoxyuridine monophosphate
GEO: Gene expression omnibus
HR: Hazard ratio
HRM: High resolution melting
HSPB1: Heat shock 27kDa protein 1
ICD-10: The international classification of diseases, version 10
MIQE: Minimum information for publication of qPCR experiments
MRPL19: Mitochondrial ribosomal protein L19
OMIM: Online mendelian inheritance in man
PCR: Polymerase chain reaction
PD: Progression of the disease
PharmGKB: The pharmacogenomics knowledgebase
POLR2A: DNA-directed RNA polymerase II subunit A
PPAT: Phosphoribosylpyrophosphate amidotransferase
PR: Partial response
PSMC4: Proteasome (prosome, macropain) 26S subunit, ATPase, 4
qPCR: Quantitative real-time PCR
RECIST: Response evaluation criteria in solid tumors
RRM1/2: Ribonuclease reductase subunit M1/2
SD: Stabilization of the disease
SLC29A1: Solute carrier transporter 29A1
Ta: Annealing temperature
TK1: Thymidine kinase
Tm: Melting temperature
TYMP: Thymidine phosphorylase
TYMS: Thymidylate synthase
UCK1/2: Uridine cytidine kinase 1/2
UICC: Union for international cancer control
UMPS: Uridine monophosphate synthetase
UPB1: Beta-ureidopropionase
UPP1/2: Uridine phosphorylase 1/2
