# Supplementary material for: Molecular profile of 5-fluorouracil pathway genes in colorectal carcinoma
Source: BMC Cancer. 2016 Oct 12;16:795. doi: 10.1186/s12885-016-2826-8 (PMC5062913; doi:10.1186/s12885-016-2826-8)

**Supplementary Table S1:** List of TaqMan Gene Expression Assays used in the study

| **Gene symbol** | **Assay ID** | **OMIM number** | **Exon boundary** | **Amplicon length** | **PCR efficiency** |
| --- | --- | --- | --- | --- | --- |
| **REFERENCE GENES** | | | | | |
| EIF2B1 | Hs00426752_m1 | 606686 | 4 - 5 | 75 | 91 |
| MRPL19 | Hs00608519_m1 | 611832 | 2 - 3 | 72 | 91 |
| POLR2A | Hs00172187_m1 | 180660 | 1 - 2 | 61 | 91 |
| PSMC4 | Hs00197826_m1 | 602707 | 6 - 7 | 83 | 92 |
| **TARGET GENES** | | | | | |
| DPYD | Hs00559279_m1 | OMIM: 612779 | 1 - 2 | 74 | 91 |
| DPYS | Hs00154808_m1 | OMIM: 613326 | 1 - 2 | 77 | NA |
| PPAT | Hs00601264_m1 | OMIM: 172450 | 1 - 2 | 68 | 87 |
| RRM1 | Hs00168784_m1 | OMIM: 180410 | 1 - 2 | 78 | 98 |
| RRM2 | Hs01072069_g1 | OMIM: 180390 | 9 - 10 | 128 | 85 |
| SLC29A1 | Hs01085706_m1 | OMIM: 602193 | 2 - 3 | 75 | 99 |
| TK1 | Hs00177406_m1 | OMIM: 188300 | 3 - 4 | 118 | 95 |
| TYMP | Hs00157317_m1 | OMIM: 131222 | 4 - 5 | 95 | 97 |
| TYMS | Hs00426591_m1 | OMIM: 188350 | 6 - 7 | 87 | 101 |
| UCK1 | Hs01075618_m1 | OMIM: 609328 | 2 - 3 | 72 | 95 |
| UCK2 | Hs00367072_m1 | OMIM: 609329 | 3 - 4 | 72 | 96 |
| UMPS | Hs00165978_m1 | OMIM: 613891 | 1 - 2 | 109 | 81 |
| UPB1 | Hs00255472_m1 | OMIM: 606673 | 1 - 2 | 59 | 100 |
| UPP1 | Hs00427695_m1 | OMIM: 191730 | 2 - 3 | 108 | 93 |
| UPP2 | Hs00542792_m1 | Gene ID: 151531 | 8 - 9 | 66 | NA |

Footnote:

NA = not applicable - PCR amplification efficiency could not be estimated due to the low expression level

**Supplementary Table S2:** Promoter CpG methylation profiling: Sequence of primers for sodium bisulfite converted DNA bases and PCR conditions

| **Primer Name** | **Promoter location*** | **Primer sequence** | **Number of** | **Amplicon** | **Tm[°C]** | **Ta[°C]** |
| --- | --- | --- | --- | --- | --- | --- |
|  |  |  | **CpGs^#^** | **length [bp]** |  |  |
| TK1_for | Chr17:78,186,200-78,189,108 | AAGGTGAGGTTATTTGAGGGTT | 8 | 140 | 61.5 | 58 |
| TK1_rev |  | TACTACCTAACTCCCCCAACAA |  |  | 61.2 |  |
| PPAT_for | Chr4:56,433,521-56,438,401 | GATGTTGTAGGGTGGAGTTAGTT | 4 | 126 | 59.9 | 56 |
| PPAT_rev |  | AAAATTAAATCCGTTACTCCCA |  |  | 60.1 |  |
| RRM1_for | Chr11:4,093,800-4,096,201 | AAATTTTTTTAGGGTTTTGATTTG | 7 | 154 | 60.1 | 56 |
| RRM1_rev |  | AACTCCAACCCAAACTCC |  |  | 58.9 |  |
| RRM2_for | Chr2:10,119,600-10,124,801 | TTAGTTTGGGTAGGGGTAAGG | 2 | 70 | 60.6 | 56-60 |
| RRM2_rev |  | ACCCTTCCCATTAACTATACCAT |  |  | 60.3 |  |
| UCK1_for | Chr9:131,530,126-131,531,601 | GAGGATATTAATAGGTGTGGATGGTT | 9 | 115 | 62.9 | 56 |
| UCK1_rev |  | AAACTCCCCCACAACCTCT |  |  | 62.4 |  |
| UCK2_for | Chr1:165,827,000-165,829,898 | TTTATGGGGGAAGGGTAGG | 3 | 81 | 62.4 | 56-60 |
| UCK2_rev |  | AAAATCCTACGAAAAACCCTCTC |  |  | 62.3 |  |
| UMPS_for | Chr3:124,729,800-124,731,770 | GTGTAGTTTTGGGGTTATTGGT | 11 | 171 | 60.4 | 59 |
| UMPS_rev |  | CCTATCCTTTCCCTTCCTAAAC |  |  | 60.7 |  |
| TYMP_for | Chr22:50,524,627-50,527,628 | TTTGGGATTAGTGGGGAGTT | 9 | 169 | 62.1 | 58 |
| TYMP_rev |  | AACTACCTCCAAAAAAACCCAC |  |  | 61.9 |  |
| UPP1_for | Chr7:48,088,000-48,090,932 | AGTAGGGAGAGGATTAGGAAAGA | 5 | 90 | 60.2 | 58 |
| UPP1_rev |  | CTACACTCTAACCCCCAAAAAC |  |  | 60.3 |  |
| UPP2_for | Chr2:157,874,600-157,877,401 | AATTTAGGATTGGTTTTATGGGT | 1 | 83 | 60.2 | 58 |
| UPP2_rev |  | ATAAAACCAAACTCAAAACCCTT |  |  | 60.5 |  |
| SLC29A1_for | Chr6:44,218,400-44,220,305 | GGTCGTTTGTTGTAGTTTTTTTT | 10 | 123 | 59.9 | 56-58 |
| SLC29A1_rev |  | AACCCCTAATTCTCTCCCTC |  |  | 60.0 |  |
| DPYS_for | Chr8:104,365,802-104,367,773 | AGGTTGGGTTGGAGTTTAA | 5 | 199 | 59.0 | 58 |
| DPYS_rev |  | TAAATTTCTTTCCCTTTAACACC |  |  | 59.0 |  |
| DPYD_for | Chr1:97,917,200-97,922,001 | TTTATTGAGTATAGGGGTTATGG | 7 | 141 | 57.6 | 56 |
| DPYD_rev |  | CCGACCCTAATCTACCTATTT |  |  | 57.7 |  |
| UPB1_for | Chr22:24,494,600-24,494,801 | GCGTGTTTTTATTTGAGTTGTTT | 12 | 189 | 61.2 | 58 |
| UPB1_rev |  | AATACTTCTCCAAACATTCCTCC |  |  | 60.9 |  |

NOTE: bp, length of PCR amplicon expressed as number of base pairs; Ta, annealing temperature of primers; Tm, melting temperature of primer.

*Promoter location according to ENSEMBL using GRCh38/hg38 assembly.

**^#^**The overall methylation of all CpG sites was taken into consideration.

**Supplementary Table S3:** Stage-adjusted Cox regression of associations between transcript levels and DFI of colorectal cancer patients from the combined testing and validation I sets

HR = hazard ratio, 95% CI = 95% confidence interval

All patients (n=92)

Gene HR 95% CI *P*-value

DPYD 1.52 0.58 – 3.85 0.397

PPAT 0.66 0.25 – 1.72 0.396

RRM1 1.12 0.44 – 2.86 0.806

**RRM2 4.17 1.35 – 12.50 0.013**

SLC29A1 1.49 0.57 – 3.85 0.415

TK1 1.72 0.64 – 4.55 0.283

TYMP 2.44 0.90 – 6.67 0.080

TYMS 2.13 0.77 – 5.88 0.144

UCK1 0.51 0.20 – 1.33 0.172

UCK2 1.10 0.43 – 2.86 0.840

UMPS 1.49 0.55 – 4.00 0.438

UPP1 0.71 0.28 – 1.82 0.482

UPB1 0.55 0.20 – 1.49 0.238

5-fluorouracil-treated patients (n=50)

Gene HR 95% CI *P*-value

DPYD 0.55 0.15 – 1.95 0.351

PPAT 2.48 0.64 – 9.62 0.190

RRM1 0.69 0.20 – 2.41 0.559

RRM2 1.95 0.49 – 7.81 0.346

SLC29A1 3.43 0.72 – 16.13 0.121

TK1 1.49 0.42 – 5.32 0.536

TYMP 2.38 0.61 – 9.25 0.211

TYMS 1.65 0.39 – 6.94 0.497

UCK1 0.91 0.25 – 3.38 0.894

UCK2 1.43 0.40 – 5.10 0.584

UMPS 1.58 0.36 – 6.85 0.541

UPP1 0.42 0.11 – 1.64 0.213

**UPB1 0.25 0.06 – 0.98 0.047**

**Supplementary Figure S1:** 5-Fluorouracil pathway gene expression levels in the studied sets of colorectal cancer patients


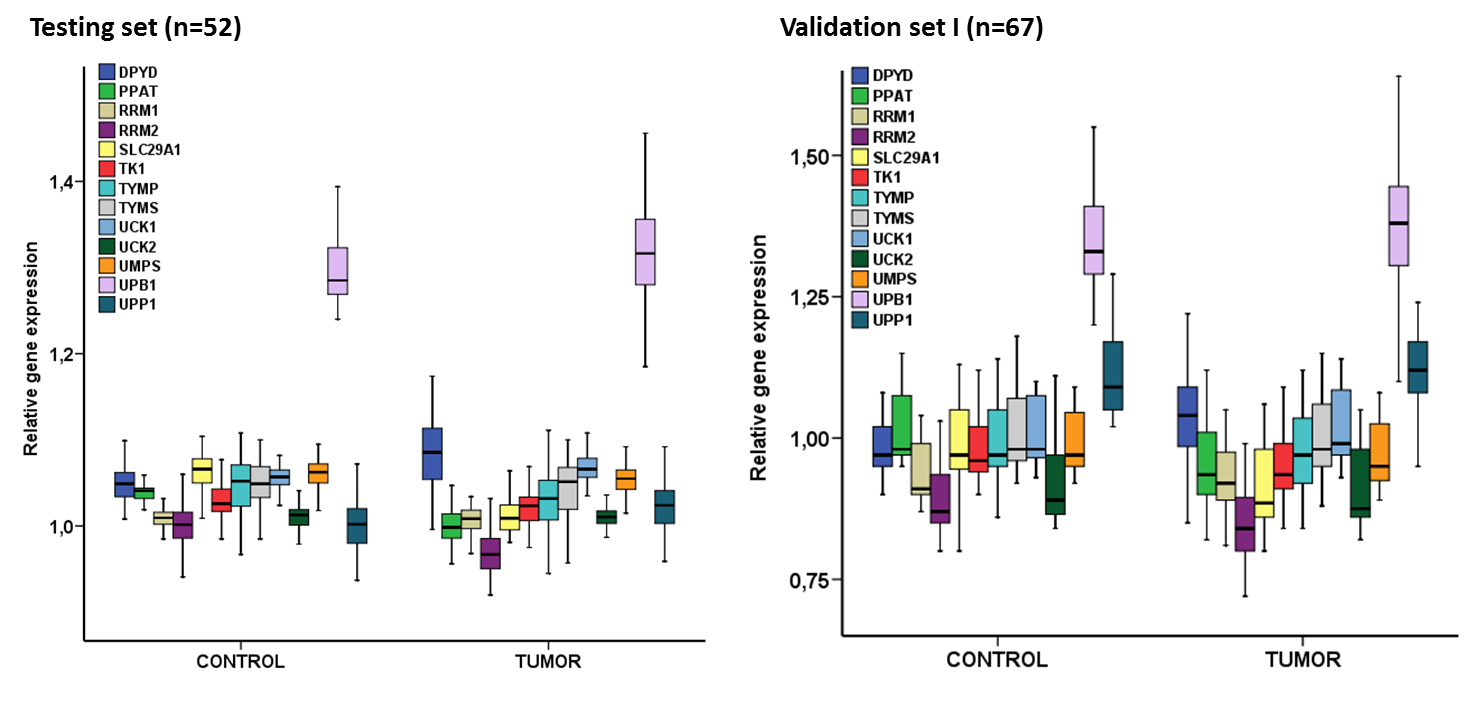


**Supplementary Figure S2:** Associations between transcript levels and DFI of colorectal cancer patients from the validation set I

DFI = disease-free survival; blue lines represent the group with lower transcript levels and green lines represent the group with higher levels than median


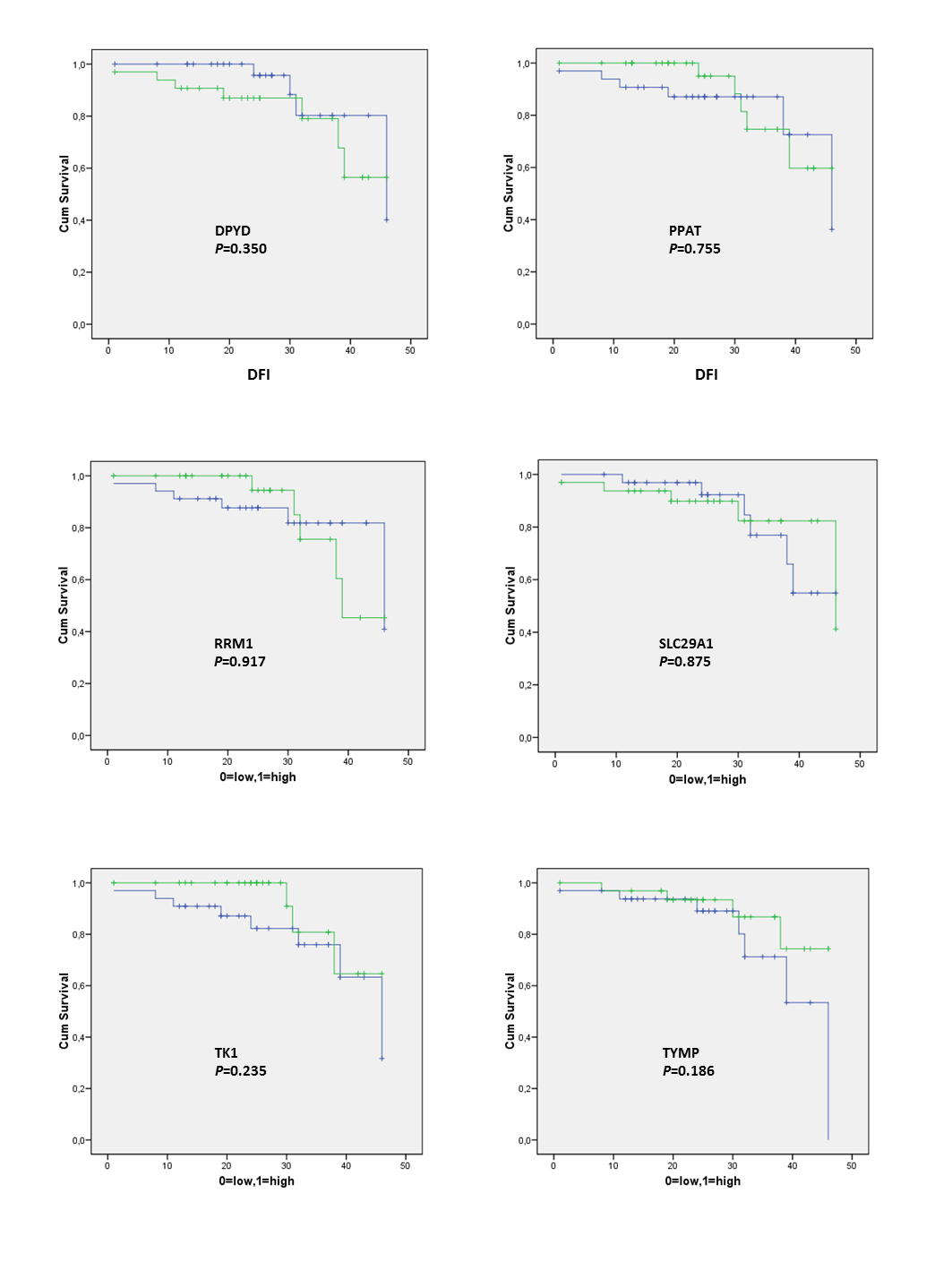


**Supplementary Figure S2:** Associations between transcript levels and DFI of colorectal cancer patients from the validation set I – continued

DFI = disease-free survival; blue lines represent the group with lower transcript levels and green lines represent the group with higher levels than median


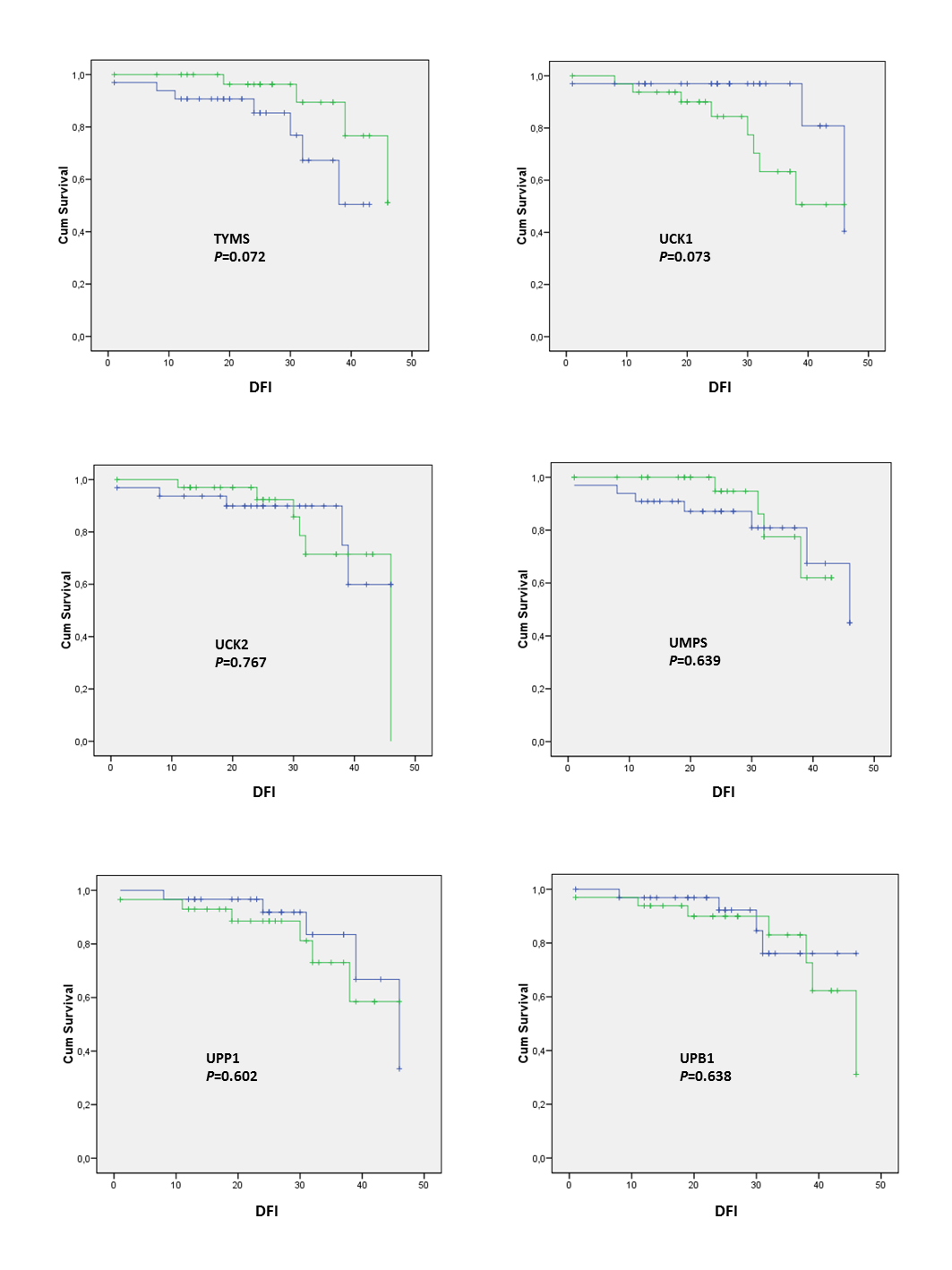


**Supplementary Figure S3:** Associations between transcript levels and DFI of colorectal cancer patients from the testing set

DFI = disease-free survival; blue lines represent the group with lower transcript levels and green lines represent the group with higher levels than median


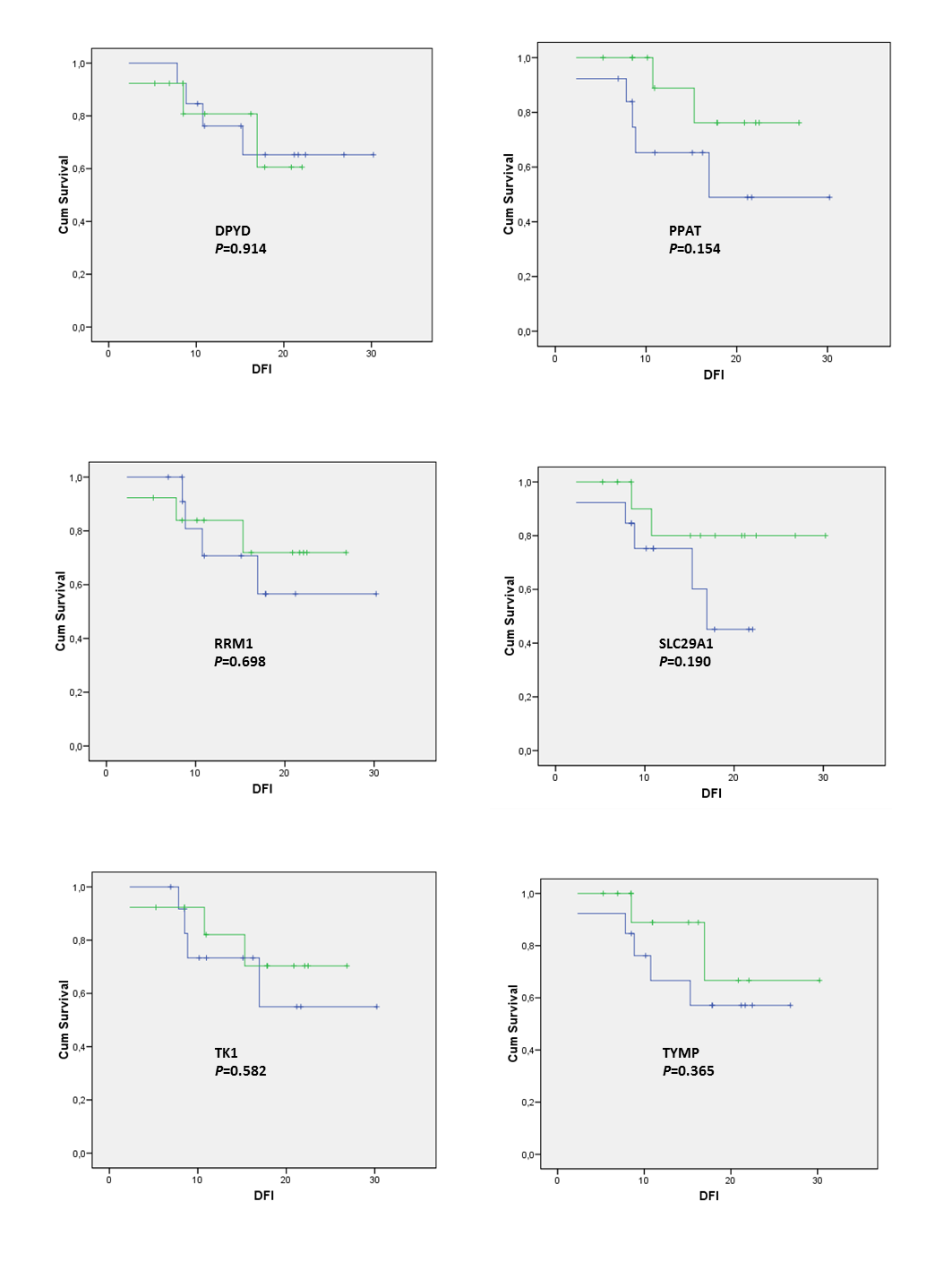


**Supplementary Figure S3:** Associations between transcript levels and DFI of colorectal cancer patients from the testing set – continued

DFI = disease-free survival; blue lines represent the group with lower transcript levels and green lines represent the group with higher levels than median


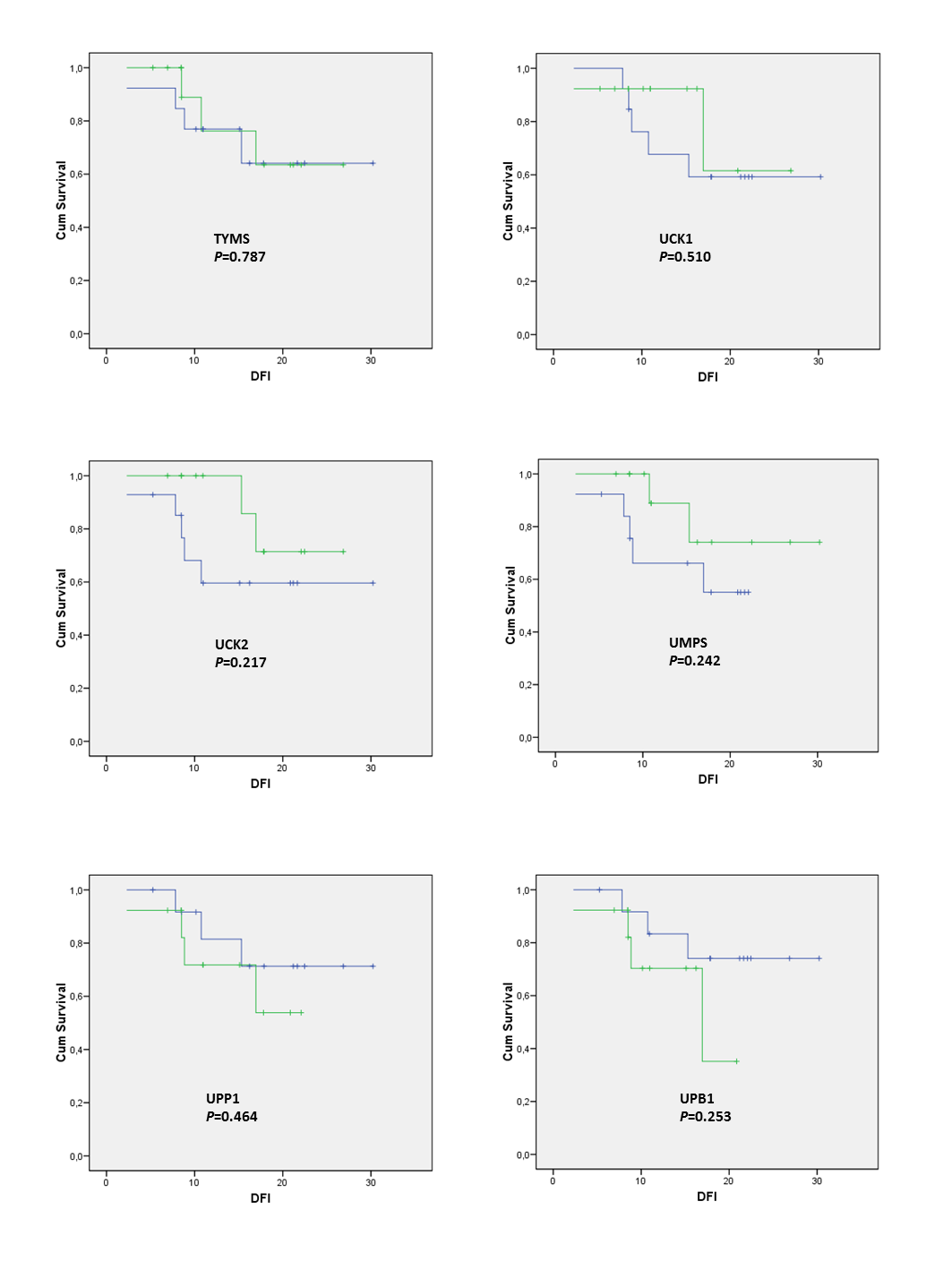


**Supplementary Figure S3:** Associations between transcript levels and DFI of colorectal cancer patients from the testing set – continued

DFI = disease-free survival; blue lines represent the group with lower transcript levels and green lines represent the group with higher levels than median


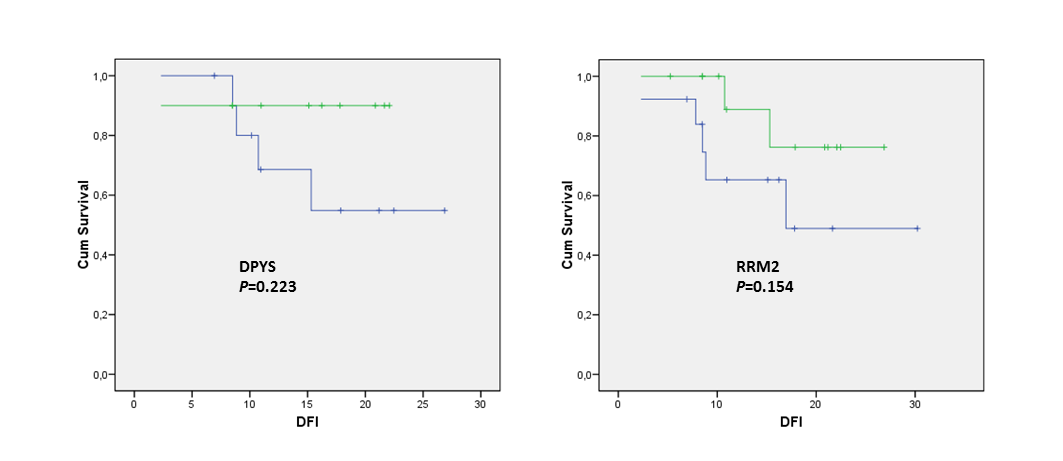


**Supplementary Figure S4:** Associations between transcript levels and DFI of colorectal cancer patients from the combined testing and validation I set

DFI = disease-free survival; blue lines represent the group with lower transcript levels and green lines represent the group with higher levels than median


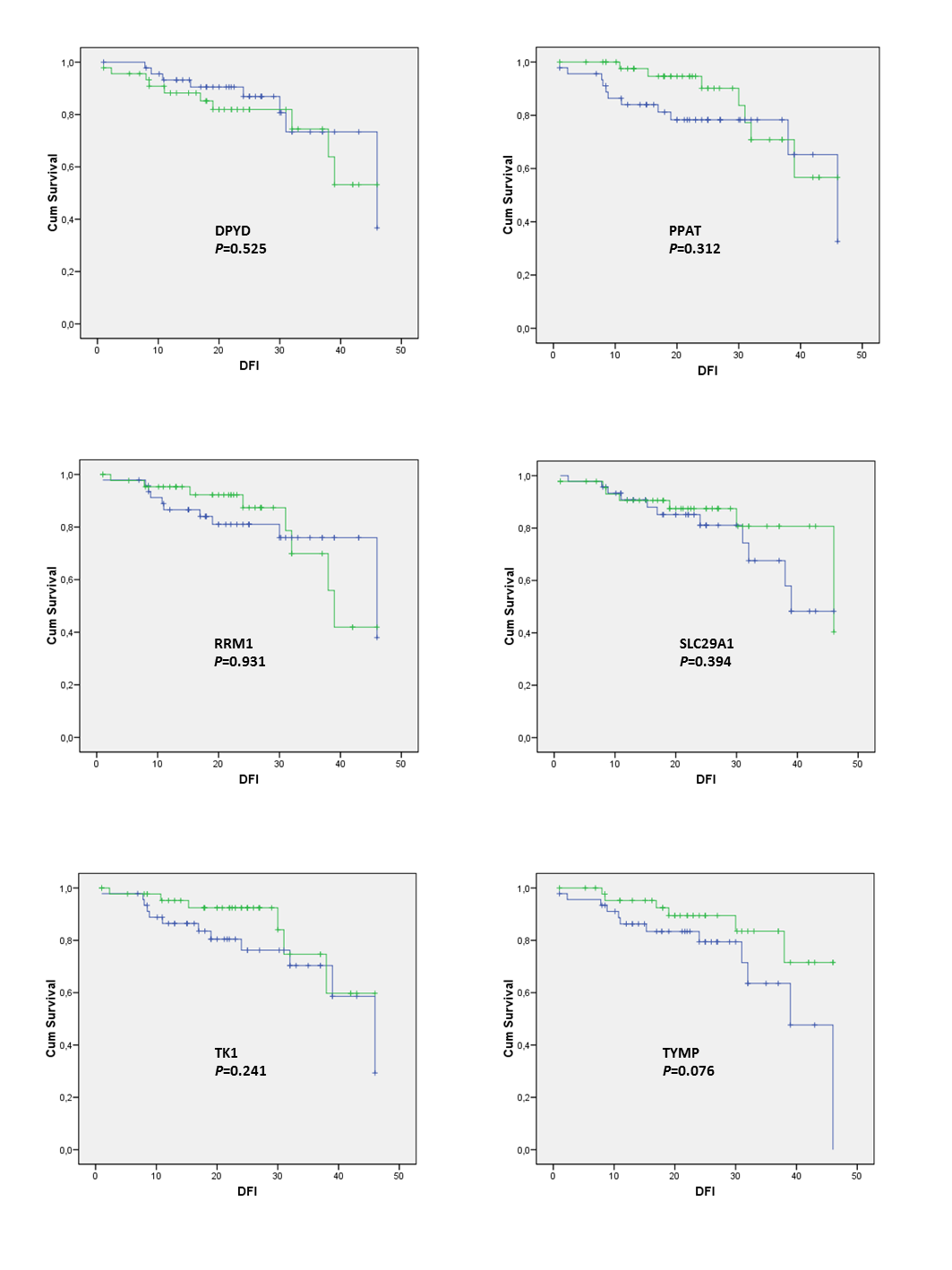


**Supplementary Figure S4:** Associations between transcript levels and DFI of colorectal cancer patients from the combined testing and validation I set – continued

DFI = disease-free survival; blue lines represent the group with lower transcript levels and green lines represent the group with higher levels than median


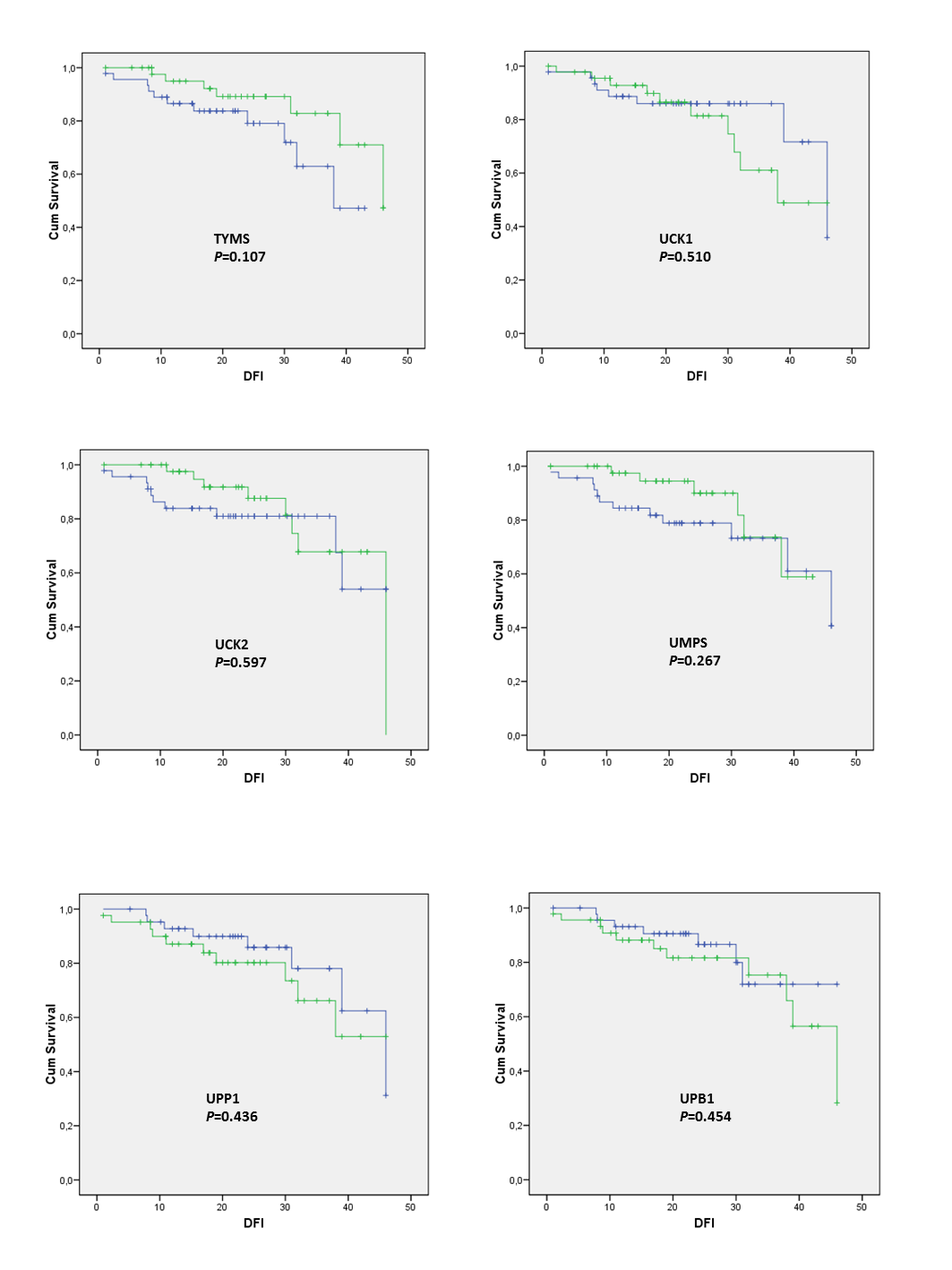


**Supplementary Figure S5:** Associations between transcript levels and DFI of 5-fluorouracil-treated colorectal cancer patients from the combined testing and validation I set

DFI = disease-free survival; blue lines represent the group with lower transcript levels and green lines represent the group with higher levels than median


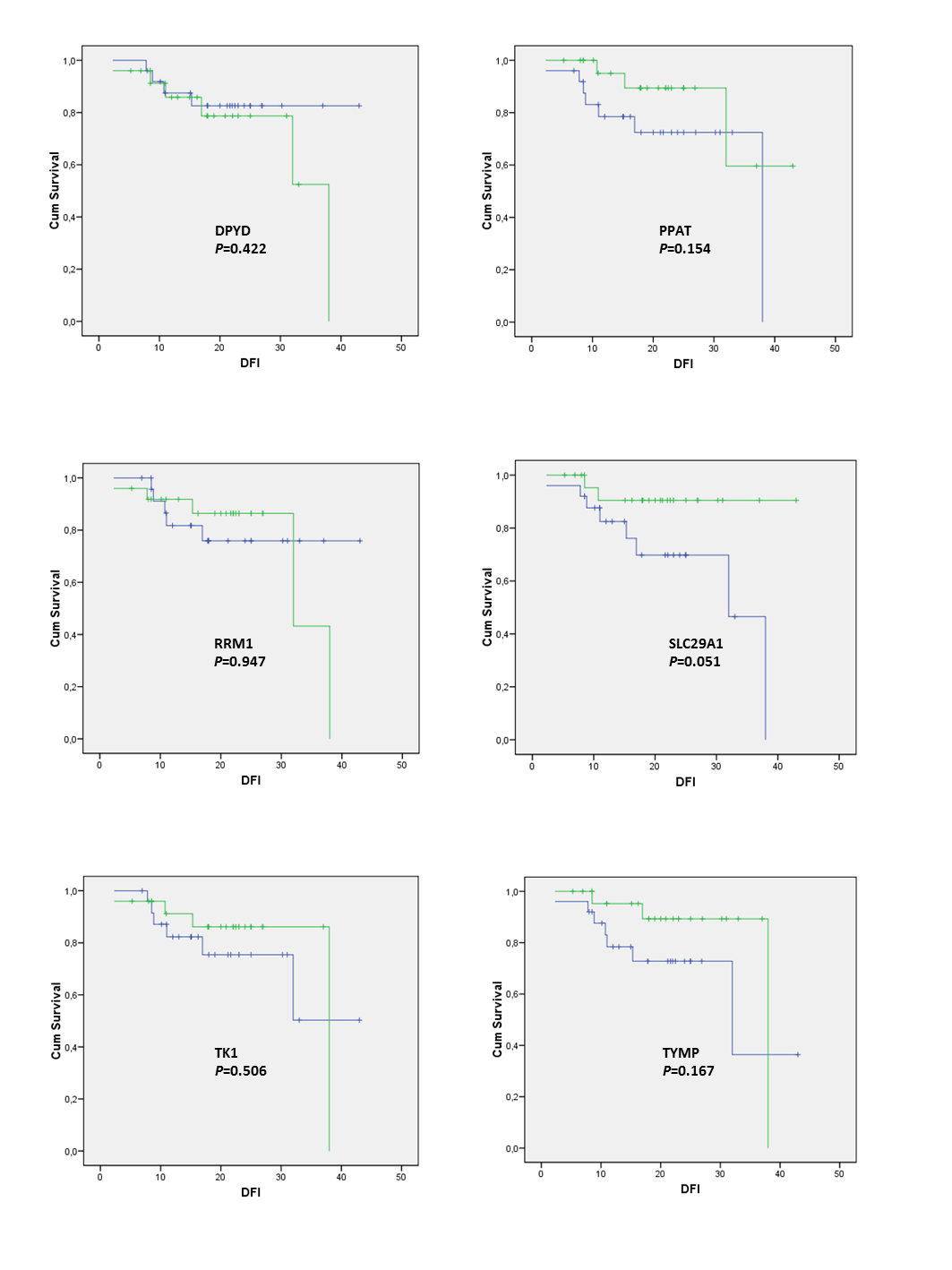


**Supplementary Figure S5:** Associations between transcript levels and DFI of 5-fluorouracil-treated colorectal cancer patients from the combined testing and validation I set – continued

DFI = disease-free survival; blue lines represent the group with lower transcript levels and green lines represent the group with higher levels than median


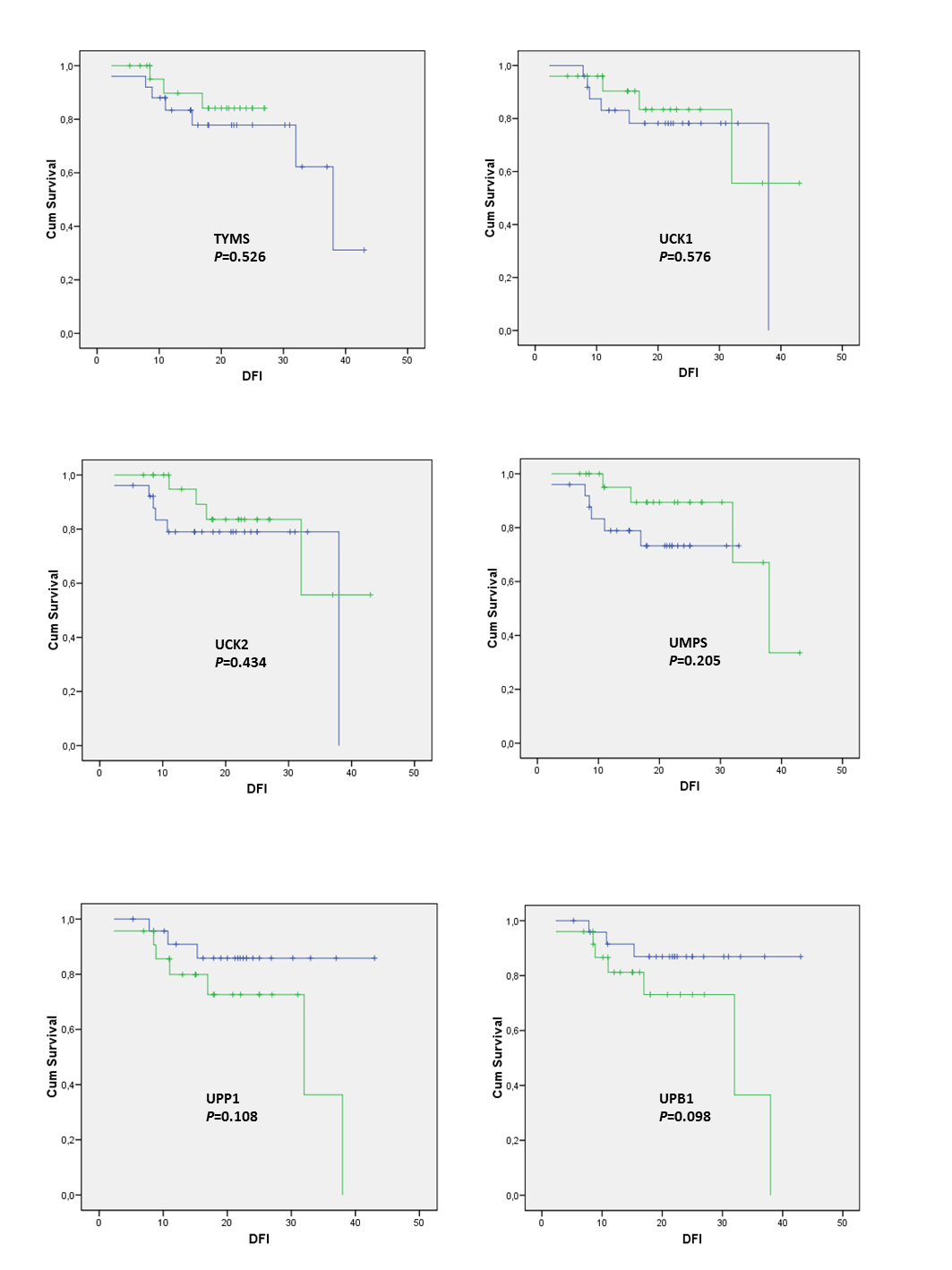


**Supplementary Figure S5:** Associations between transcript levels and DFI of 5-fluorouracil-treated colorectal cancer patients from the combined testing and validation I set – continued

DFI = disease-free survival; blue lines represent the group with lower transcript levels and green lines represent the group with higher levels than median


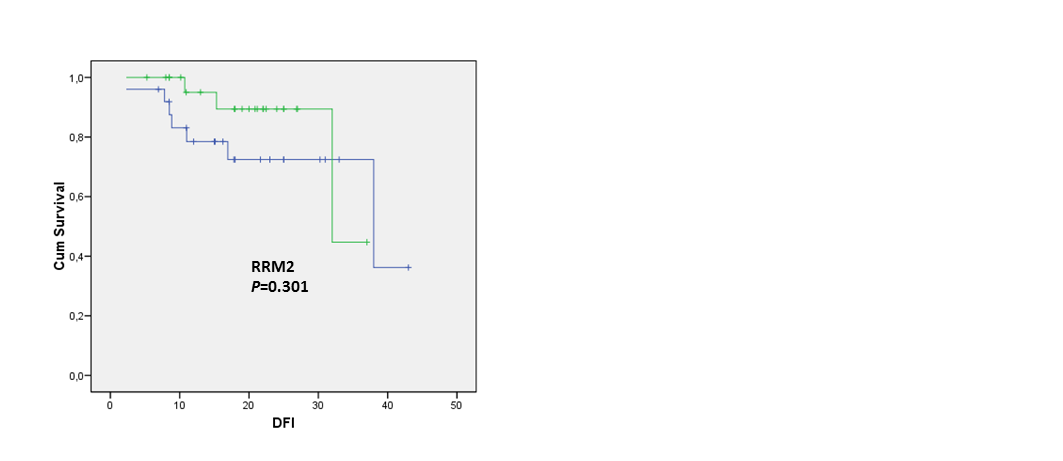


**Supplementary Figure S6:** Associations between transcript levels and DFI of untreated colorectal cancer patients from the validation I set

DFI = disease-free survival; blue lines represent the group with lower transcript levels and green lines represent the group with higher levels than median


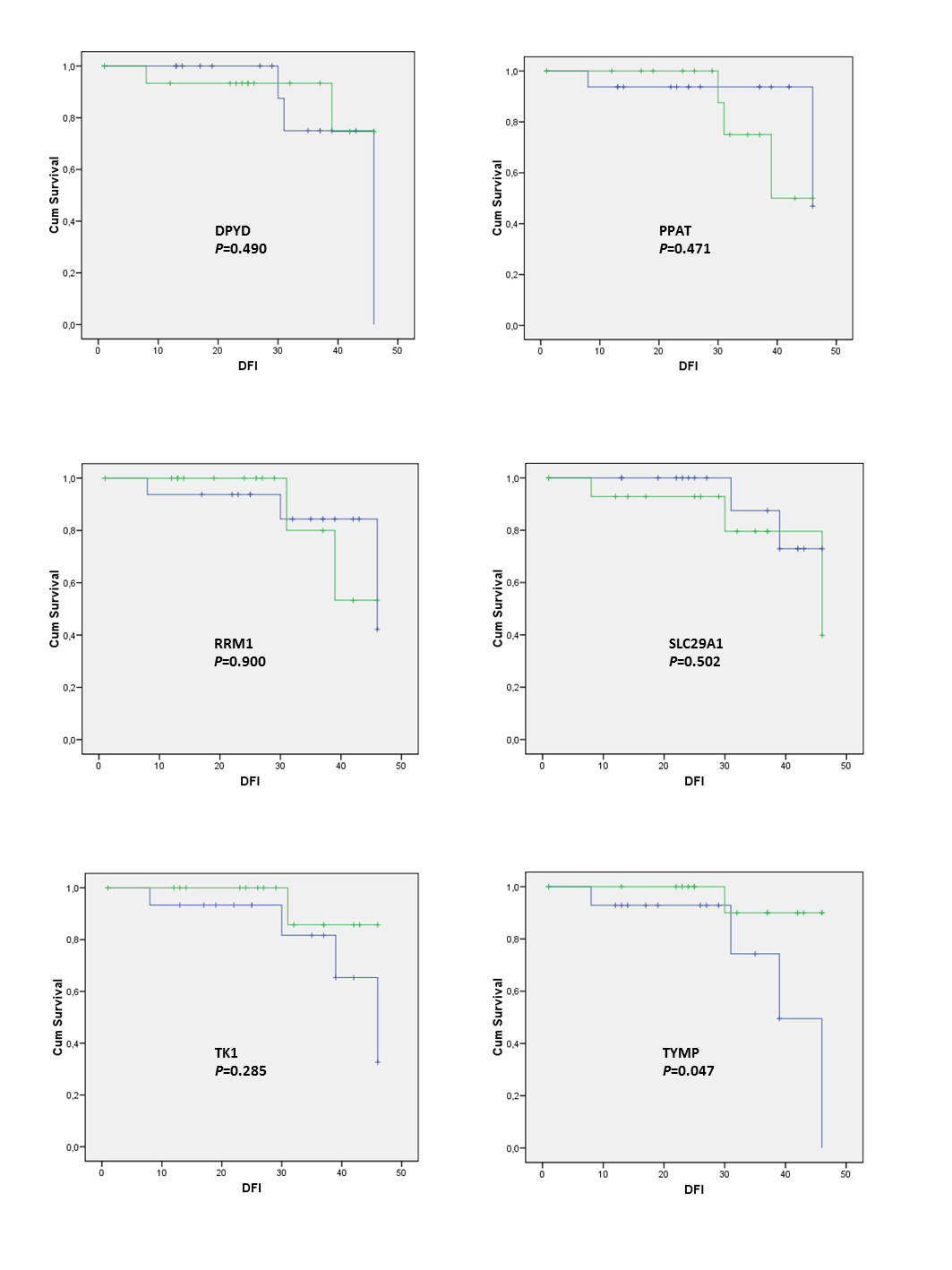


**Supplementary Figure S6:** Associations between transcript levels and DFI of untreated colorectal cancer patients from the validation I set – continued

DFI = disease-free survival; blue lines represent the group with lower transcript levels and green lines represent the group with higher levels than median


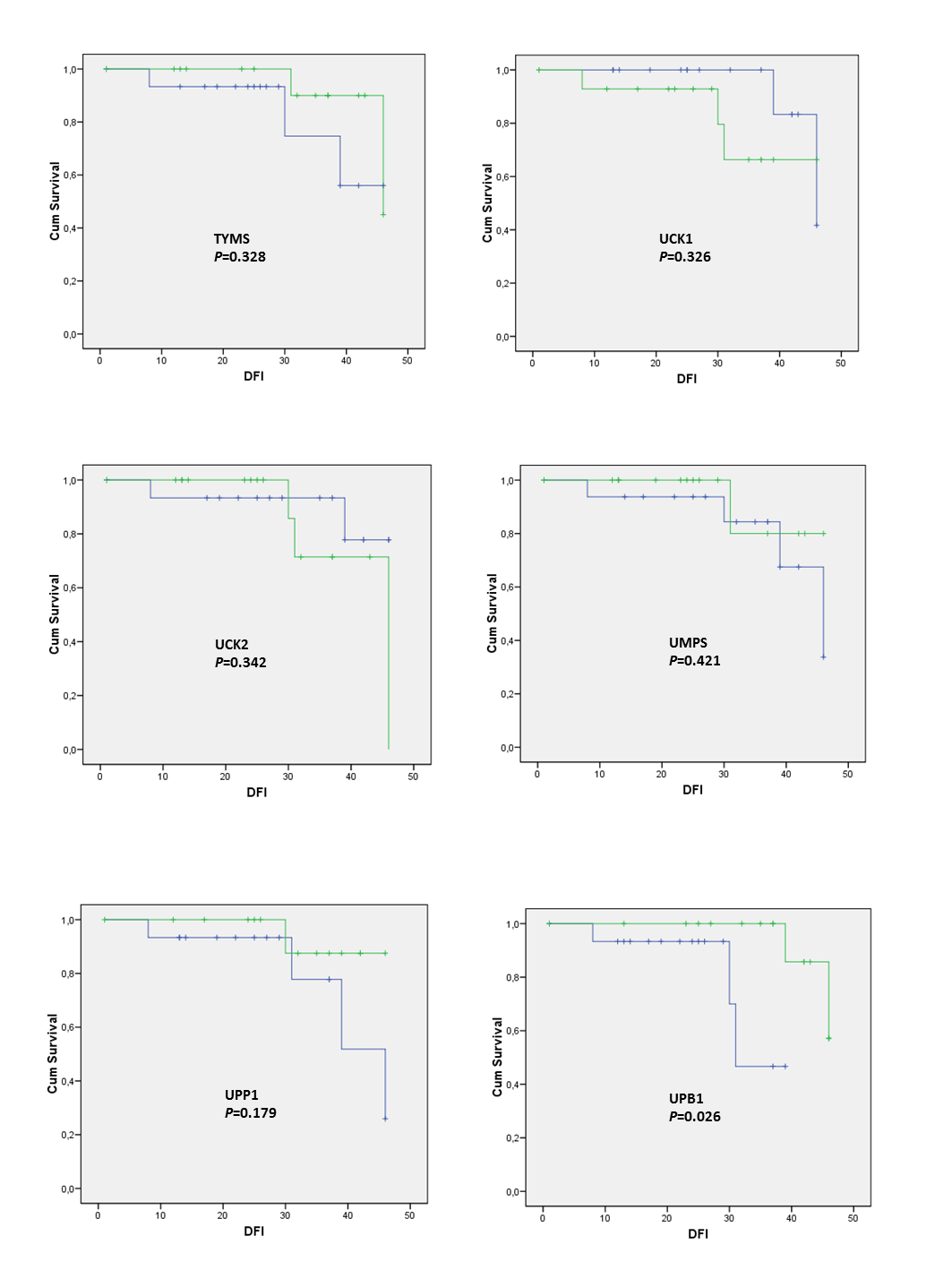


**Supplementary Figure S6:** Associations between transcript levels and DFI of untreated colorectal cancer patients from the validation I set – continued

DFI = disease-free survival; blue lines represent the group with lower transcript levels and green lines represent the group with higher levels than median


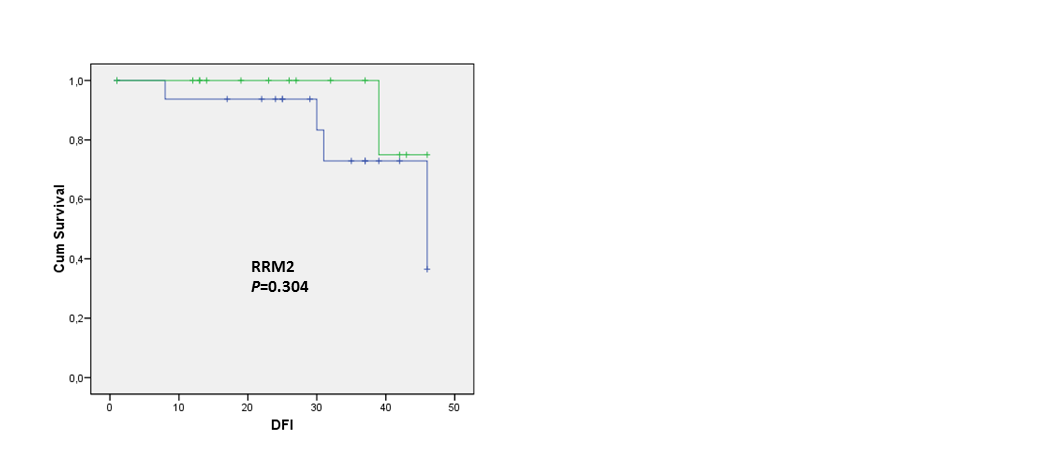


**Supplementary Figure S7:** Association between *UPB1* methylation levels and DFI of colorectal cancer patients


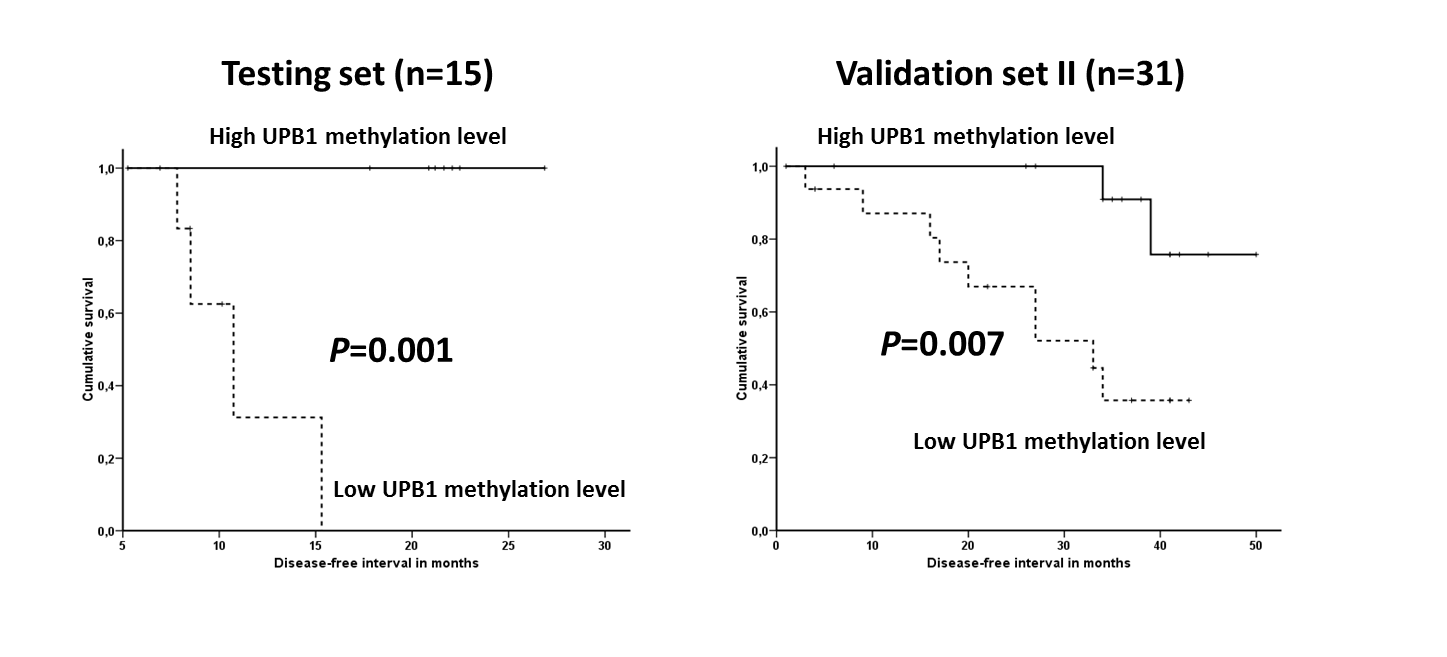


Kaplan-Meier survival curves were plotted for patients from the testing (n=15, seven stage IV patients excluded) and validation II (n=31) sets. Patients were divided into two groups according to the median of intratumoral gene methylation levels. Dashed lines represent the group with lower methylation levels and solid lines represent the group with higher levels than median. Differences between groups were compared using Log-rank test.

**Supplementary Figure S8:** Association of RRM2 expression with disease-free survival of colorectal cancer patients from GEO database

Analysis was performed using SurvExpress (Aguirre-Gamboa et al. 2013) with intratumoral gene expression and clinical data for 947 patients from GSE12945 set from Gene Expression Omnibus (GEO). Disease-free survival (upper plot, *P*=0.050 by the Log Rank test and *P*=0.052, HR=1.42, 95% CI=0.99-2.03 by the Cox regression model) was analyzed using disease recurrence risk data (high risk=high RRM2 expression in green and low risk=low RRM2 expression in red, lower plot).


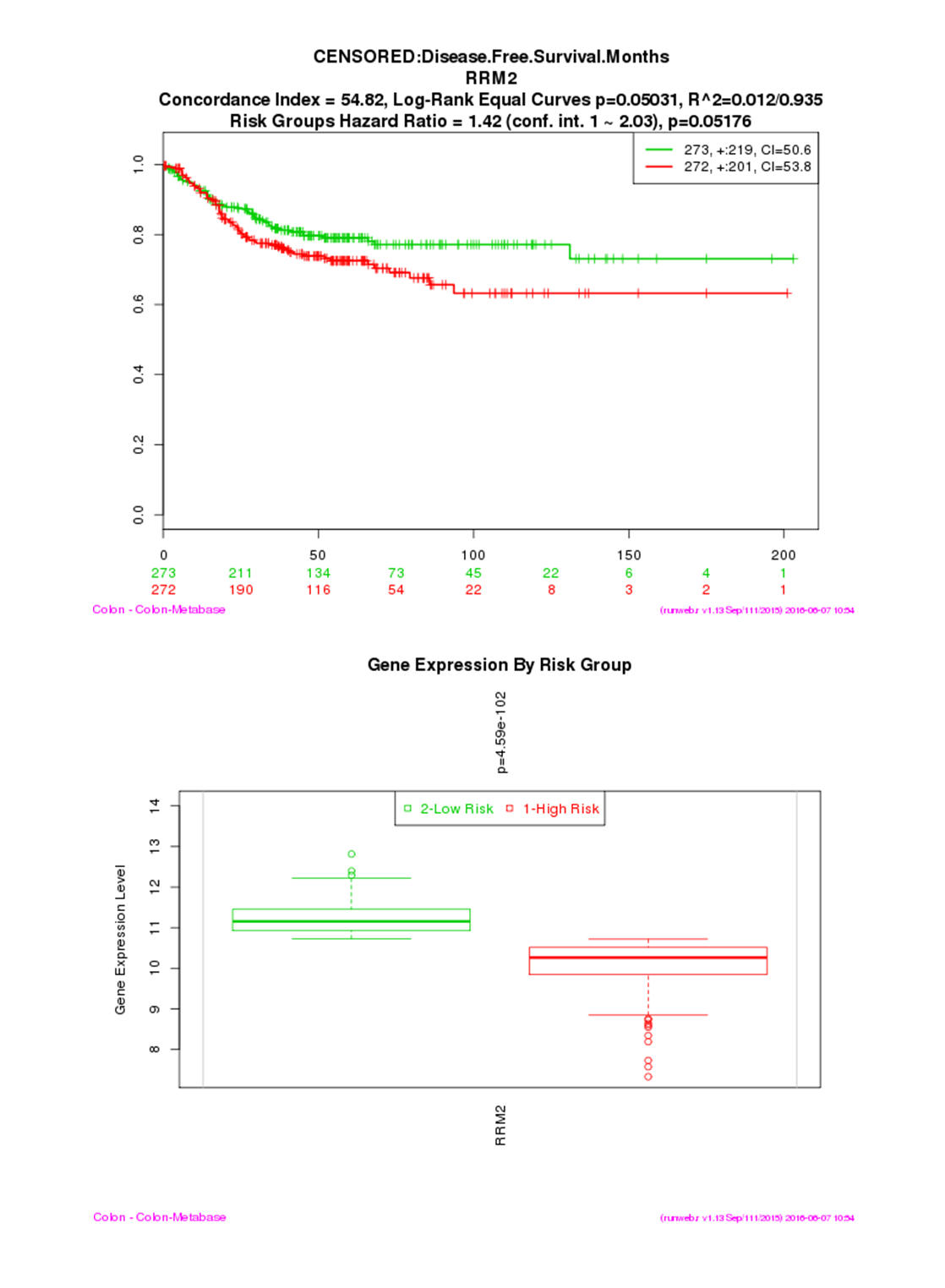


Aguirre-Gamboa R, et al. SurvExpress: An Online Biomarker Validation Tool and Database for Cancer Gene Expression Data Using Survival Analysis. *PLoS ONE* 2013; 8(9): e74250.

**Supplementary Figure S9:** Methylation profiles of 5-FU pathway genes in human colorectal tumor (red boxes) and mucosa (green boxes) tissues from MethHC database[35]

Footnotes: COAD=samples from colon adenocarcinomas, READ=samples from rectal adenocarcinomas, *P<0.05, **P<0.005


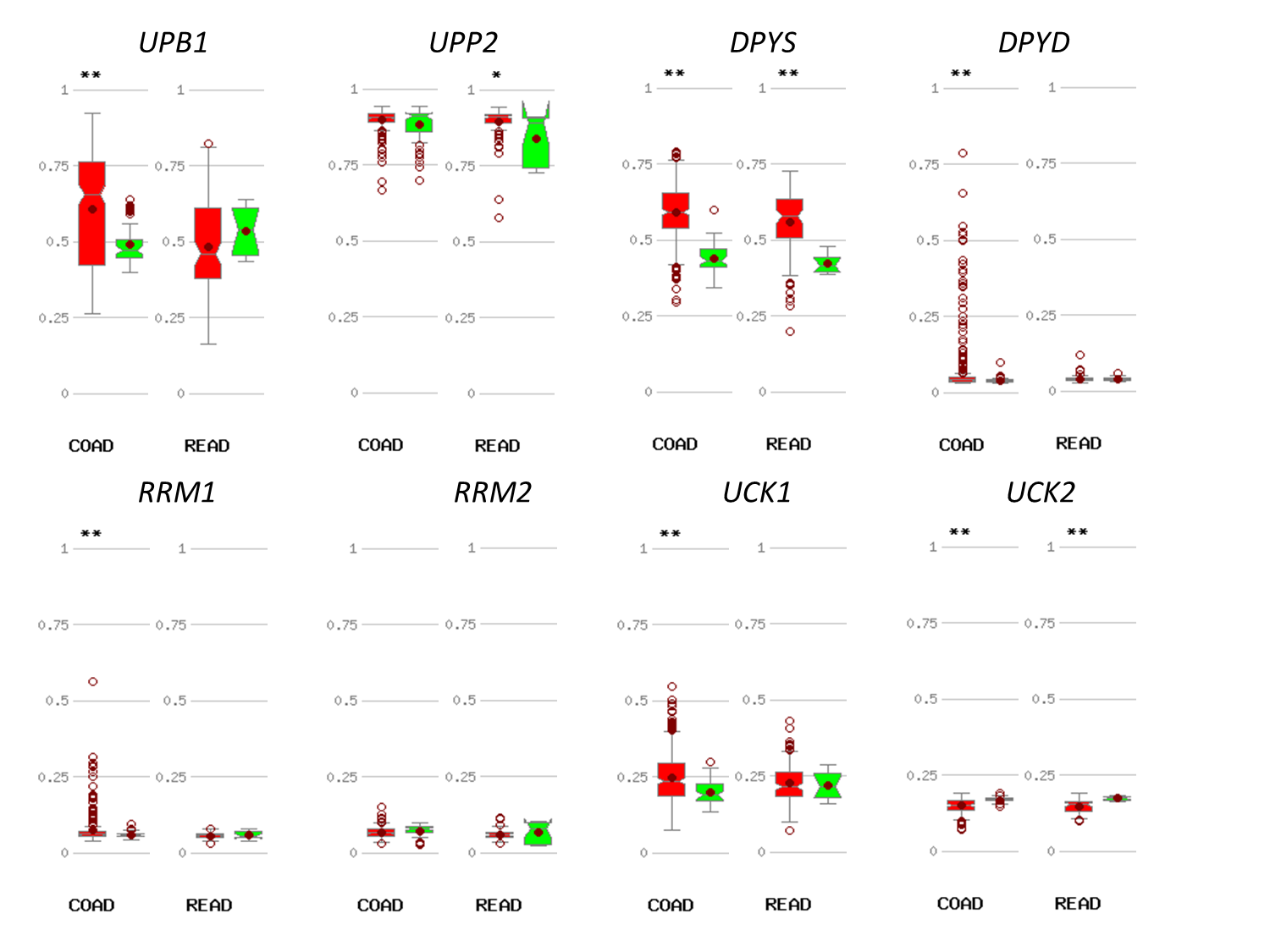


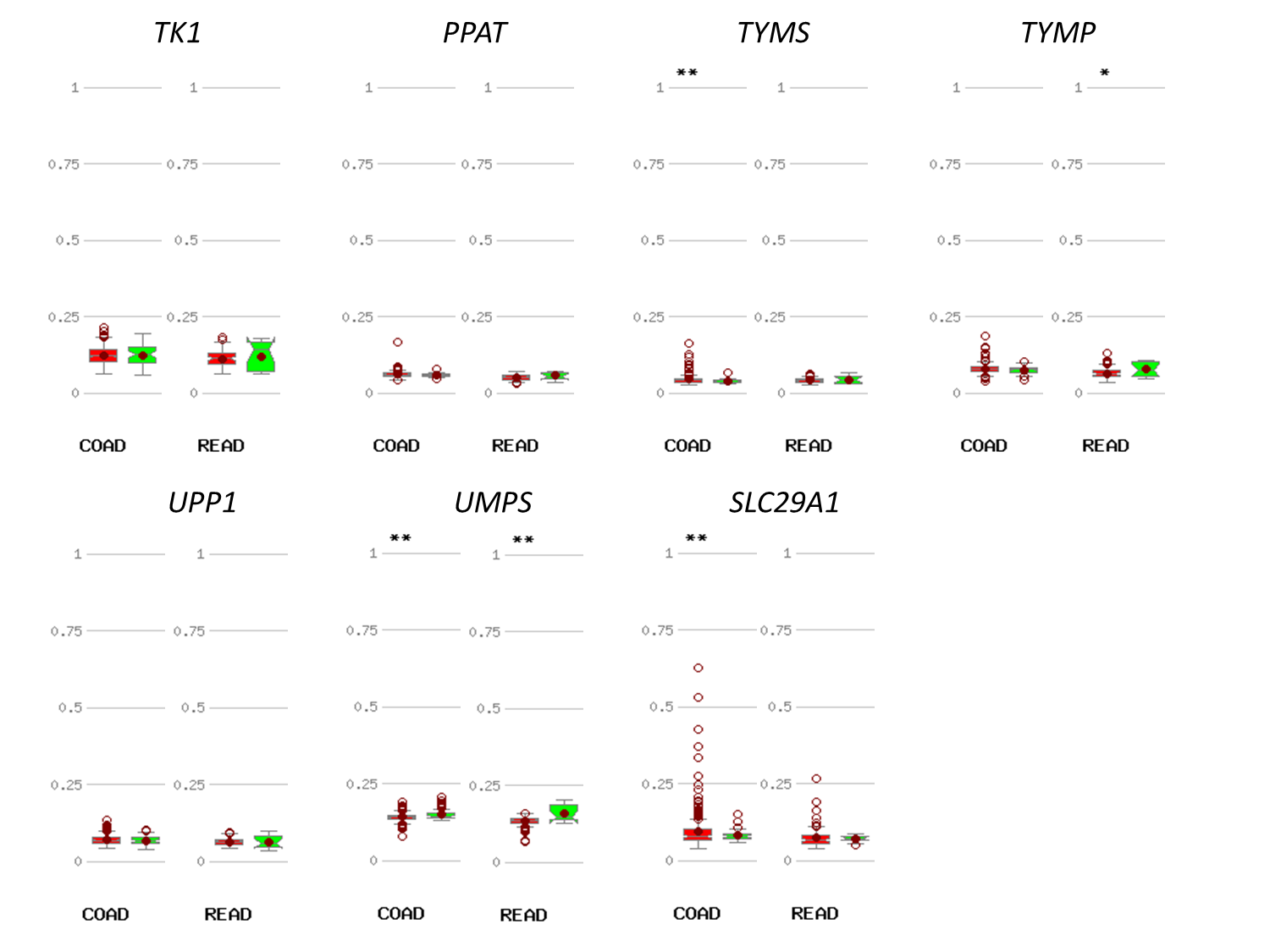

Supplement: Additional file 1: Table S1. — Lists TaqMan Gene Expression Assays used in the study. Table S2 shows sequence of primers and PCR conditions used for promoter CpG methylation profiling. Table S3 shows results of stage-adjusted Cox regression of associations between transcript levels and DFI of colorectal cancer patients from the combined testing and validation I sets. Figure S1 depicts 5-Fluorouracil pathway gene expression levels in the studied sets of colorectal cancer patients. Figure S2 shows results of analysis of associations between transcript levels and disease-free survival of colorectal cancer patients from the validation set I. Figure S3 shows results of analysis of associations between transcript levels and disease-free survival of colorectal cancer patients from the testing set. Figure S4 shows results of analysis of associations between transcript levels and disease-free survival of colorectal cancer patients from the combined testing and validation I set. Figure S5 shows results of analysis of associations between transcript levels and disease-free survival of 5-fluorouracil-treated colorectal cancer patients from the combined testing and validation I set. Figure S6 shows results of analysis of associations between transcript levels and disease-free survival of untreated colorectal cancer patients from the validation I set. Figure S7 shows results of analysis of associations between UPB1 methylation levels and disease-free survival of colorectal cancer patients. Figure S8 shows analysis of association of RRM2 expression with disease-free survival of colorectal cancer patients based on publicly available GEO database. Figure S9 shows analysis of methylation profiles of 5-FU pathway genes in human colorectal tumor (red boxes) and mucosa (green boxes) tissues from publicly available MethHC database. (DOC 1916 kb) [file 12885_2016_2826_MOESM1_ESM.doc]
